# An Overview of Analytical Methods for Quantitative Determination of Coenzyme Q10 in Foods

**DOI:** 10.3390/metabo13020272

**Published:** 2023-02-14

**Authors:** Andersina Simina Podar, Cristina Anamaria Semeniuc, Simona Raluca Ionescu, Maria-Ioana Socaciu, Melinda Fogarasi, Anca Corina Fărcaș, Dan Cristian Vodnar, Sonia Ancuța Socaci

**Affiliations:** 1 Department of Food Science, University of Agricultural Sciences and Veterinary Medicine of Cluj-Napoca, 3-5 Mănăştur St., 400372 Cluj-Napoca, Romania; 2 Centre for Technology Transfer-BioTech, 64 Calea Florești, 400509 Cluj-Napoca, Romania; 3 Department of Food Engineering, University of Agricultural Sciences and Veterinary Medicine of Cluj-Napoca, 3-5 Mănăştur St., 400372 Cluj-Napoca, Romania

**Keywords:** coenzyme Q10, foods, extraction, quantification, wastes, recovery

## Abstract

Food analysts have developed three primary techniques for coenzyme Q10 (CoQ10) production: isolation from animal or plant matrices, chemical synthesis, and microbial fermentation; this literature review is focused on the first method. Choosing the appropriate analytical method for determining CoQ10 in a particular food product is essential, as this analyte is a quality index for healthy foods; various associations of extraction and quantification techniques are available in the literature, each having advantages and disadvantages. Several factors must be considered when selecting an analytical method, such as specificity, linear range, detection limit, quantification limit, recovery rate, operation size, analysis time, equipment availability, and costs. In another train of thought, the food sector produces a significant amount of solid and liquid waste; therefore, waste-considered materials can be a valuable source of CoQ10 that can be recovered and used as a fortifying ingredient or dietary supplement. This review also pursues identifying the richest food sources of CoQ10, and has revealed them to be vegetable oils, fish oil, organs, and meat.

## 1. Introduction

The coenzyme Q term refers to a class of homologous quinones found in various living organisms, including microorganisms, plants, animals, and humans, that share a benzoquinone ring structure with an isoprenoid side chain of varying lengths [1,2]. The isoprenyl tail length of CoQ is species-specific, containing between 6 and 10 subunits [3,4]; in humans, higher plants, and mammals, it comprises ten isoprene units, hence the name of coenzyme Q10 (CoQ10) [2,5,6]. As for microorganisms, the isoprene chain contains fewer subunits, such as six (CoQ6) in *Saccharomyces cerevisiae*, seven in *Crucianella maritima* (CoQ7), or eight in *E. coli* (CoQ8) [4,7].

CoQ10 is sensitive to physicochemical changes; for example, in a basic medium and upon exposure to light or temperature (>55 °C), it becomes unstable and transforms [8,9,10]. Therefore, thermal processing destroyed it in foods [11]. CoQ10 has a molecular weight of 865 g/mol and a melting point of 49 °C [12]. In addition, it exhibits hydrophobicity (due to the polyprenyl side chain), which makes CoQ10 hard to dissolve in water and polar solvents, but easy to dissolve in nonpolar ones and lipids [8]. However, because of its higher molecular mass (863.7 Da) and poor water solubility, the absorption of CoQ10 for oral administration is limited [7,13,14,15].

Over the last decades, CoQ10 has gotten much attention, given that it is the only endogenously synthesized lipid-soluble antioxidant in cells [16,17,18]. Although CoQ10 functions like a vitamin, since it is synthesized in the human body is not considered one [12,19,20,21]. Chemically, CoQ10 is 2,3-dimethoxy-5-methyl-6-decaprenyl-1,4-benzoquinone, where Q denotes the quinone chemical group [5,10,22,23]. It occurs in two redox forms, oxidized (ubiquinone-10) and reduced (ubiquinol-10), and its normal functioning consists of continuous inter-conversion between these [24,25,26]. In tissues, the oxidized form of CoQ10 coexists with its reduced one (CoQ10H2), the latter being predominant in living beings [27,28]. Since more than 90% of CoQ10 content in human serum and biological tissues exists in the reduced form [7], ubiquinol-10 is responsible for the antioxidant properties observed in vivo [29,30]. However, ubiquinol-10 is unstable and quickly oxidizes into ubiquinone-10 in the air [17,29,31]; in addition, the latter may increase in tissues, to a certain extent, due to oxidative stress caused by the transport of animals and the following slaughter procedure [3,32,33].

Coenzyme Q is present in plants and animals, yet its amount is the highest in animal-origin products [8,34]. Flesh foods (meat, poultry, and fish) are the richest sources of dietary CoQ10 [5,6,35]; organs, such as the heart, liver, kidney, brain, pancreas, and spleen, also have elevated levels [8,19,36,37]. In addition, considerable amounts of CoQ10 are present in eggs and bee pollen [6,8,38] and, regarding vegetable-origin products, in oils and nuts [7,37,39]. Milk and dairy products are not generally considered good sources of this molecule, despite being of animal origin [39].

Food waste is a well-known issue; a third of foods produced for human use worldwide are lost or wasted [40]. Since wastes are potential sources of CoQ10, this molecule can be extracted and used in many applications, such as personal care, cosmetics, foods, beverages, feeds, pharmaceuticals, and nutraceuticals. In addition, proactive consumers now seek cognitive health products, including dietary supplements [41]. Consequently, there is a high demand for natural extracts in the food and nutraceutical industries [42]. Therefore, manufacturers of natural extracts are interested in knowing which raw materials are rich in CoQ10 and its most efficient extraction procedure from a particular matrix to maximize production [43,44,45].

In our survey of scholarly knowledge on this topic, we searched online databases for studies investigating the level of CoQ10 in vegetable and animal matrices to identify those with the highest level. As a result, information collected on tested matrices, analytical methods used, and contents of CoQ10 found are centralized in Table 1 for products of vegetable origin, in Table 2 for animal-origin ones, and discussed in Section 2 of the manuscript. This review provides an update on available analytical methods for the quantitative determination of CoQ10 in foods (Section 3); the authors also discuss the contents of CoQ10 found in vegetable- and animal-origin products, highlighting the advantages and disadvantages of the methods that reported the highest levels for a specific matrix (Section 3.1).

## 2. Biological Role and Health Benefits of CoQ10

Contrary to other lipophilic antioxidants, CoQ10 originates both from endogenous synthesis and dietary intake; whilst dietary uptake affects plasma concentrations, the rate of local endogenous synthesis predominantly influences the content of CoQ10 in tissues [3]. CoQ10 is produced in the body with the benzoquinone ring generated from phenylalanine or tyrosine and the polyprenyl side chain from acetyl-CoA through the mevalonate pathway. Infections, stress, and bad dietary habits affect the organism’s ability to synthesize it [49]. With age, the CoQ10 production rate declines [25,27,35,37].

Via its benzoquinone head group’s capacity to embark on an ongoing redox cycle, CoQ10 exerts two primary physiological functions: it relocates electrons for ATP (an essential component of respiration) production in the electron transport chain and acts as a lipophilic antioxidant (in its reduced form) by preventing the oxidation of proteins, polyunsaturated fatty acids, and DNA [25,35,50,51,52]. CoQ10 is found in the central hydrophobic region of the phospholipid bilayer that makes up the mitochondrial membrane. Here, it participates in the electron transport chain process by accepting electrons from reducing equivalents produced by breaking fatty acids and glucose and transferring them to acceptors. A proton gradient is generated when electrons move from one complex to another. As a result, adenosine triphosphate (ATP) is produced from the energy released when protons return to mitochondria [19].

CoQ10 deficiency is rare because its level in the human body is mainly maintained by endogenous synthesis; however, some drug treatments and pathophysiological conditions result in suboptimal CoQ10 levels [25]. Since its deficiency has been related to aging processes and several diseases, such as cancer, heart failure or sarcopenia, CoQ10 has gained wide popularity among researchers [21,53]. It has been shown to boost energy levels, stimulate the immune system, act as a free radical scavenger, prevent premature skin aging, and combat cardiovascular and neurodegenerative diseases [9,11,27,49,51]. Other benefits associated with CoQ10 include potentially aiding in the control of diabetes and post-cardiac surgery recovery [17,35].

Because it is fat-soluble, CoQ10 is better absorbed when consumed with an oil- or fat-rich meal [54]. However, given that the dietary contribution of CoQ10 is negligible, with daily intakes around 3 to 5 mg, in cases of deficiency, supplementation is needed [53]. Generally, levels between 100 and 200 mg/day of CoQ10 are recommended to achieve a beneficial effect, although, for treating chronic diseases, up to 1200 mg/day can be used [35].

## 3. Analytical Methods for CoQ10 Determination in Foods and Contents Found

In this review, an attempt was made to centralize data from the existing literature on the content of CoQ10 in food matrices to highlight the primary sources of CoQ10 in products of vegetable (Table 1) and animal origin (Table 2), respectively; simultaneously, we sought data on the methods of extraction and quantification of CoQ10 from these matrices.

Extraction is essential for separating a targeted molecule from a sample before its instrumental analysis towards quantifying the analyte [55]. Previous studies have reported five extraction methods (see Table S1) for determining CoQ10 in food matrices (see Table S1: 1. direct extraction method; 2. ultrasonic extraction method; 3. saponification extraction method; 4. solid-phase extraction (SPE) method; and 5. accelerated solvent extraction (ASE) method) and nine quantification methods (see Table S2: 1. HPLC (high-performance liquid chromatography) with UV (ultraviolet) detection (275 nm); 2. HPLC with DA (diode array) detection (274 and 275 nm); 3. HPLC with EC (electrochemical) detection; 4. HPLC/ESI (electrospray ionization)–MS (mass spectrometry) detection; 5. HPLC-ESI-MS/MS detection; 6. HPLC with AEC (amperometric electrochemical) detection; 7. HPLC with APCI (atmospheric pressure chemical ionization)–MS detection; 8. CEFS (Cary Eclipse fluorescence spectrometer) detection (585 and 627 nm); and 9. DPV (differential pulse voltammetry) using an electrochemical workstation). The data collected regarding CoQ10 contents found in tested vegetable- and animal-origin products and the parameters of analytical methods used for their determination are shown in Table 1 and Table 2. Here, they are presented on food categories such as herbs, vegetables, fruits, grains and seeds, and vegetable oils as regards the products of vegetable origin, and meat and poultry, fish and seafood, fish oils, eggs, milk and dairy, and bee products for those of animal origin.

The analytical methods used for CoQ10 quantification in products of vegetable origin are detailed in Table 1. In 2006, Zu et al. [46] used an LC-MS/MS method (see Figure S1) with multiple reaction monitoring for CoQ10 analysis in tobacco leaves. This method involved sample ultrasonication in anhydrous ethanol for 15 min and extraction of the supernatant with hexane, followed by the separation on an RP18 reversed-phase column (5 µm particle size, 3.9 mm ID × 150 mm L; Symmetry Shield). Low limits of detection and quantification, of 1.2 ng/mL and 4.0 ng/mL, and a measuring range of 8.4–540.0 ng/mL were obtained for this method using tandem MS/MS detectors. The recovery rates of CoQ10 were close to 100%, and the corresponding relative standard deviations (RSDs) were below 2.4%. A few years later, Stiff et al. [18] proposed a method (see Figure S2) for the routine analysis of CoQ10 in green leaves of *Nicotiana tabacum* (tobacco) using HPLC with UV detection, which consisted of direct extraction with 2-propanol and chromatographic separation on a Luna C18(2) column (4.6 mm ID × 250 mm L; Phenomenex). The sample preparation protocol involved fewer steps than the method of Zu et al. [46], using a single solvent in a small volume and a small sample amount; since the UV detector is less sensitive than MS, the LOD and the working range were poorer, at 0.063 µg/mL and 0.158–10.14 µg/mL. Surprisingly, the level of CoQ10 found by Stiff et al. [18] in tobacco leaves was higher than that reported by Zu et al. [46], most likely due to differences in the sample maturity at harvesting, as explained by the authors.

Kubo et al. [30] developed a method (see Figure S3) that simultaneously detects the reduced and oxidized forms of CoQ10; it employs direct extraction, using 2-propanol, and HPLC quantification using a system equipped with an EC detector and an RC-10 reduction column (4.0 mm ID × 15 mm L; Shiseido). Food items from groups such as herbs (parsley, perilla, and rape-leaf), vegetables (broccoli, cabbage, potato, cucumber, spinach, mustard spinach, eggplant, radish, onion, garlic, lotus root, pea, bean, soybean, asparagus, and avocado), fruits (orange, apple, strawberry, grapefruit, banana, and persimmon), seeds (almond), oils (sesame oil and soybean oil), meat products (pork meat, beef meat, chicken meat, beef liver, and chicken heart), fish and seafood products (mackerel flesh, horse mackerel flesh, sardine flesh, five-ray yellowtail flesh, young yellowtail flesh, cod flesh, salmon flesh, tuna flesh, flatfish flesh, scallop flesh, oyster flesh, cuttlefish flesh, octopus flesh, and shrimp flesh), egg products (hen’s egg), and milk and dairy products (milk, yoghurt, and cheese) were successfully analyzed using this method. Over the range of 0.040–50 ng/injection, which corresponded to 0.08–100 mg/g or 0.16–200 mg/g of the analytes in foods, the ECD response was linear.

The usefulness of a new approach for the automatic determination of CoQ10 in different food samples (see Figure S4), based on the use of magnetoliposomes (MLs) containing hydrophobic magnetic gold nanoparticles and the long-wavelength fluorophore cresyl violet, has been demonstrated by Román-Pizarro et al. [21]. MLs were concentrated just before the detector, a CEFS, using a flow system and an external electromagnet. The subsequent introduction of Triton X-100 and CoQ10 caused MLs lysis and the cresyl violet oxidation, decreasing the fluorescence signal. This method has been satisfactorily applied in the analysis of parsley, spinach, avocado, peanut, pistachio, pork liver, and beef liver. A mixture of ethanol/*n*-hexane (2:5, *v*/*v*) was used to extract CoQ10 directly from each sample before the fluorescence spectrometry. The method reached an LOD (0.008 µmol/L) comparable to that reported by Rodríguez-Acuña et al. [27] (0.001 µmol/L), who performed solid-phase extraction and LC-MS analysis, but lower than those values obtained by the LC-UV methods of Mattila and Kumpulainen [24] (0.58 µmol/L), Souchet and Laplante [17] (0.14 µmol/L), and Xue et al. [38] (0.12 µmol/L); the recovery rates ranged from 83.5% to 101.3%, similar to those obtained using the LC-UV techniques.

Li et al. [28] advanced a DPV method (see Figure S5) that uses an electrochemical workstation equipped with three electrodes (a silver disc as the working electrode, a platinum column counter electrode, and a saturated calomel reference electrode) to determine CoQ10 in food matrices. Samples of fish (sardine flesh and saury flesh), animal tissues (pork meat, pork heart, pork liver, pork kidney, beef meat, and chicken meat), and vegetables (broccoli, tomato, carrot, corn, spinach, pea, orange, kiwi, apricot, cherry, peanut, rapeseed, and barley) were directly extracted with a nitrogen-saturated ethanol/water (95:5, *v*/*v*) phosphate-buffered saline (PBS) solution, of pH 6.5, before measurements. The calibration curve was linear over the concentration range of 0.0863–863 mg/kg, the LOD was 0.0288 mg/kg, and recovery rates of spiked samples were between 91 and 108%. When they used an HPLC-UV method to analyze the same food matrices, the results were in good agreement with those obtained using the DPV method.

The analytical method used by Mattila and Kumpulainen [24] for the quantification of CoQ10 in different food items involved either direct solvent extraction (see Figure S6) or saponification before extraction (see Figure S7), followed by HPLC-DAD analysis on a 201TP54-C18 column (5 µm particle size, 4.6 mm ID × 250 mm L; Vydac) fitted with an ODS guard column. The detector response was linear in the 12–500 ng/injection range; the LOD reached 5.0 ng/injection, and the mean recovery rate was 93%. The direct ethanol/*n*-hexane (2:5, *v*/*v*) extraction was employed for most of the samples taken in this study, such as cauliflower, potato, tomato, carrot, pea, bean, orange, clementine, apple, blackcurrant, lingonberry, strawberry, reindeer meat, pork heart, beef meat, beef heart, chicken meat, pollack flesh, hen’s egg, skimmed milk (1.5% fat), and yoghurt, because it was simple to perform and efficient. However, this procedure was unsuitable for rapeseed oil, pork liver, beef liver, Emmental cheese, and Edam cheese, resulting in interfering compounds that made quantification difficult; therefore, these samples were accurately dosed using saponification with an aqueous potassium hydroxide solution before the *n*-hexane extraction. Some authors [8] advise avoiding saponification as it constitutes a primary source of analytical errors. In addition, if the employed chemical conditions are not appropriate, alkaline hydrolysis can cause considerable destruction of CoQ10. Therefore, alcoholic potassium hydroxide or sodium hydroxide solutions are usually used in the saponification reaction to eliminate this problem. However, ethanol is not suitable for preparing these alkaline solutions since, in the alkaline environment, it reacts with CoQ10, producing some ethoxy artifacts. Thus, to protect the analyte against chemical destruction, pyrogallol or ascorbic acid is recommended to be added.

In another study, Weber et al. [16] determined CoQ10 in tomato, carrot, cucumber, orange, apple, kiwi, hen’s egg, yoghurt, hard cheese, and cream cheese using a direct extraction method and HPLC-UV analysis (see Figure S8). This entailed sample homogenization in a saline solution and triplicate extraction with ethanol/hexane (1:5, *v*/*v*), followed by the chromatographic separation of the extract on an ODS column (5 µm particle size, 4 mm ID × 300 mm L; Spherisorb).

Al-Faraji and Shanshal’s [47] protocol (see Figure S9) to isolate and quantify CoQ10 in Iraqi dates consisted of sample saponification using a methanolic solution of pyrogallol and an aqueous solution of potassium hydroxide followed by triplicate extraction with petroleum ether. First, the saponified extract was purified by column chromatography (over an alumina column). Then, the separation of CoQ10 from the purified solution was performed by TLC (thin-layer chromatography) on a silica gel F_254_ glass plate. Finally, an HPLC instrument equipped with a UV detector and an S5 ODS2 column (5 μm particle size, 4.6 mm ID × 250 mm L; Spherisorb) was used to quantify CoQ10 in the collected fraction.

The study of Pyo [7] investigated the concentrations of CoQ10 and CoQ9 in several commercial vegetable oils commonly consumed in Korea, namely, sesame oil, maize germ oil, perilla oil, grape seed oil, and soybean oil. Their analytical method (see Figure S10) employed saponification before solvent extraction and quantification by HPLC/ESI-MS detection. An aqueous pyrogallol solution and a sodium hydroxide solution were used for saponification. After saponification of the oil sample, ubiquinones were extracted three times with *n*-hexane. Then, the chromatographic separation was performed on a Poroshell 120 EC-C18 column (2.7 µm particle size, 3.0 mm ID × 50 mm L; Agilent Technologies).

Rodríguez-Acuña et al. [27] aimed to develop and optimize a simple and fast analytical method (see Figure S11) for quantifying CoQ9 and CoQ10 in vegetable oils. This method was successfully applied, with an LOQ of 0.025 mg/kg for both compounds, to soybean, rapeseed, and sunflower oils, and involved the isolation of the coenzyme Q fraction by solid-phase extraction (SPE) on amino phase eluting with heptane/ethyl ether (80:20, *v*/*v*), evaporation of the organic solvent under nitrogen, dissolution of residue in acetonitrile/tetrahydrofuran (90:10, *v*/*v*), and finally, analysis by RP (reverse-phase)-HPLC/APCI-MS on an Xterra MS RP C18 column (3.5 µm particle size, 2.1 mm ID × 50 mm L; Waters) fitted with an Xterra RP C18 guard (2.1 mm ID × 10 mm L; Waters). The method’s sensitivity was based on the highly efficient formation of CoQ9 and CoQ10 radical anions by negative atmospheric pressure ionization. In addition, interferences were minimized by mass detection of the [M¯˙] ions (*m*/*z* = 797.5 for CQ9 and *m*/*z* = 862.5 for CQ10) using a triple-quadrupole mass spectrometer in selective reaction monitoring mode.

The analytical methods used for CoQ10 quantification in products of animal origin are detailed in Table 2. In 2014, Tobin et al. [35] quantified the CoQ10 in pork and beef meat using a modified version of the solvent extraction method (see Figure S12) described by Mattila and Kumpulainen [24] and HPLC with UV detection on a Nucleosil 100-5 C18 column. The extraction method involved sample treatment with Hanks′ balanced salt solution (HBSS), homogenization of the digestate with ethanol and *n*-hexane in a ratio of 2:5 (*v*/*v*), followed by centrifugation. The top layer of *n*-hexane was saved, and the lower layer was re-extracted twice using ethanol and *n*-hexane. Finally, the collective *n*-hexane solution was rotary-evaporated, and the residue was dissolved in 2-propanol.

Niklowitz et al. [3] determined the CoQ10 content in pork meat, pork heart, pork liver, pork kidney, and pork brain via a direct extraction method (see Figure S13) using 2-propanol as the homogenization medium and hexane for extraction in a ratio of 3:1 (*v*/*v*), followed by HPLC-EC analysis on a column ProntoSIL 120-3-C18-SH PEEK (Bischoff Analysentechnik und Geräte). Their method was linear (12–60 mg fresh muscle tissue/sample), sensitive (200 pmol CoQ10/sample), and reproducible (RSDs of 6.0 and 3.2% for total CoQ10 within-day and day-to-day, respectively), with a mean recovery rate for the total CoQ10 of 94%.

The research by Mattila et al. [31] pursued a comparison of in-line-connected DA and EC detectors in the RP-HPLC analysis of CoQ9 and CoQ10 in pork heart, beef meat and Baltic herring flesh extracted with *n*-hexane/ethanol (5:1, *v*/*v*), using the slightly modified procedure of Weber et al. [16] (see Figure S14), using a 201TP54-C18 column (5 µm particle size, 4.6 mm ID × 250 mm L; Vydac). The RSDs of CoQ9 and CoQ10 contents found in these samples were less than 10%, regardless of the detector used, be it DA or EC. The detection systems’ responses were linear in the evaluated range, 10–200 ng/injection, with correlation coefficients exceeding 0.999. The recovery rates of added coenzymes Q9 and Q10 ranged between 73 and 105% for the DAD and from 74 to 103% for the EC detector. CoQ9 and CoQ10 had detection limits of 4 and 6 ng/injection using DA detection, and of 0.2 and 0.3 ng/injection by EC detection, respectively. The two detecting systems’ results were generally similar. Although the EC detector was 20-fold more sensitive than the DA detector, in some cases, the selectivity was poorer.

The assay of CoQ10 employed by Purchas et al. [22] (see Figure S15) to analyze beef meat, beef heart, beef liver, and lamb meat was that of Mattila et al. [31], using an HPLC-UV system fitted with a C18-reverse-phase column. Differently, the *n*-hexane/ethanol ratio of the mixture used for extraction was 5:2 (*v*/*v*) in this study.

In the study of Ercan and El [5], the extraction and analysis of CoQ10 in beef meat, beef heart, and beef liver was performed using the solvent extraction method published by Mattila and Kumpulainen [24] (see Figure S12) on HPLC apparatus equipped with a UV detector.

A simple and efficient extraction procedure followed by fast RP-HPLC with DA detection (see Figure S16) has been optimized and validated by Souchet and Laplante [17] to determine the level of CoQ10 in mackerel flesh, herring flesh, mackerel heart, and herring heart. In the first step, the sample treatment consisted of homogenization with sodium dodecyl sulphate (SDS) and a sodium chloride solution followed by extraction with ethanol/hexane (2:5, *v*/*v*). Then, an HPLC analysis of fish extracts was carried out on a 201TP54-C18 column (5 µm particle size, 4.6 mm ID × 250 mm L; Vydac) fitted with an ODS guard column. No purification step was necessary before chromatographic analysis. The method validation revealed excellent sensitivity (2.5 ng/injection), reproducibility (RSDs of 1.5–1.6%) and recovery (100.3–105.1%). In 2009, Laplante et al. [48] used the same method (see Figure S16) to measure the CoQ10 content in whole mackerel and whole herring. As for mackerel and herring oils that were also tested, they were extracted by enzymatic hydrolysis from lyophilized fish samples using Protamex™ and supercritical CO_2_ (SCO_2_), then directly dissolved in 2-propanol before HPLC analysis; the results show that the highest content of CoQ10 was obtained in mackerel oil when applying extraction by enzymatic hydrolysis, while in herring oil by supercritical carbon dioxide extraction using the following conditions: 600 g CO_2_/h and 5% EtOH.

Mandrioli et al. [9] evaluated the content of CoQ10 in Italian whole cow milk by sample saponification with an ethanolic potassium hydroxide solution and a pyrogallol solution, followed by extraction with petroleum ether/diethyl ether (9:1, *v*/*v*) (see Figure S17). An HPLC system equipped with a DA detector and a Poroshell 120 EC-C18 column (2.7 µm particle size, 3.0 mm ID × 50 mm L; Agilent Technologies) was used for chromatographic separation of the resulting extract. Although CoQ10 is a liposoluble constituent, the contents they found in whole raw milk were not statistically correlated with the sample fat content.

Manzi and Durazzo [39] developed a chromatographic method (see Figure S18) to determine CoQ10 in cheeses rapidly. First, samples of Provola cheese, Pecorino cheese, and Bagoss cheese were subjected to saponification with a potassium hydroxide solution and an ethanolic pyrogallol solution, followed by extraction with hexane/ethyl acetate (9:1 *v*/*v*); finally, an HPLC-UV analysis on a Kromasil silica column (5 µm particle size, 4.6 mm ID × 250 mm L; Phenomenex) fitted with a SecurityGuard cartridge precolumn (with silica phase; Phenomenex) was carried out. The working range was linear between 0.810 and 2.025 μg CoQ10/mL; the LOD found was 0.024 μg/mL, and the LOQ was 0.069 μg/mL.

A method for determining CoQ10 in bee pollen (rape bee pollen, apricot bee pollen, tea bee pollen, and mixed bee pollen) was developed by Xue et al. [38] (see Figure S19) by applying an online cleanup of accelerated solvent extraction (ASE) and using absolute ethanol, an environmentally acceptable organic solvent. The chromatographic analysis of bee pollen extracts was carried out on an HPLC instrument fitted with a UV detector and an Eclipse XDB C18 column (5 µm particle size, 4.6 mm ID × 150 mm L; Agilent Technologies). The assay was linear over the 0.25–200 mg/L concentration range, the LOD was 0.16 mg/kg, and the LOQ of 0.35 mg/kg; intra- and inter-day RSDs were under 6.3%, and recovery rates exceeded 90%.

Contents of CoQ10 in products of vegetable origin (Table 1) ranged between 2.1 and 27.6 µg/g for herbs (11.5–27.6 µg/g in tobacco-green leaf, 7.5–11.4 µg/g in parsley, 2.1 µg/g in perilla, 6.7 µg/g in rape-leaf); 0.08 and 24.3 µg/g for vegetables (7.0–11.3 µg/g in broccoli, 2.7–6.6 µg/g in cauliflower, 3.8 µg/g in cabbage, 0.5–1.6 µg/g in potato, 0.19–2.6 µg/g in tomato, 0.24–4.8 µg/g in carrot, 0.08 µg/g in cucumber, 4.4–5.1 µg/g in corn, 0.44–13.5 µg/g in spinach, 2.0 µg/g in mustard spinach, 1.0 µg/g in eggplant, 0.70 µg/g in radish, 0.90 µg/g in onion, 3.5 µg/g in garlic, 0.96 µg/g in lotus root, 2.3–3.3 µg/g in pea, 1.8–2.3 µg/g in bean, 6.8 µg/g in soybean, 2.2 µg/g in asparagus, 9.5–24.3 µg/g in avocado); 0.49 and 21.1 µg/g for fruits (1.0–3.9 µg/g in orange, 0.90 µg/g in clementine, 1.1–1.3 µg/g in apple, 3.4 µg/g in blackcurrant, 0.90 µg/g in lingonberry, 0.50–1.4 µg/g in strawberry, 1.3 µg/g in grapefruit, 0.80 µg/g in banana, 0.49–2.6 µg/g in kiwi, 0.80 µg/g in persimmon, 4.1–4.6 µg/g in apricot, 12.2–14.5 µg/g in cherry, 21.1 µg/g in dry date); 3.0 and 25.5 µg/g for grains and seeds (5.0 µg/g in almond, 11.5–25.5 µg/g in peanut, 18.5–22.2 µg/g in pistachio, 3.0–3.2 µg/g in rapeseed, 8.2–9.7 µg/g in barley); and 1.3 and 97.6 µg/g for vegetable oils (1.3 µg/g in olive oil, 17.6–31.5 µg/g in sesame oil, 17.7 µg/g in maize germ oil, 84.9 µg/g in perilla oil, 20.2 µg/g in grape seed oil, 53.8–97.6 µg/g in soybean oil, 46.4–63.5 µg/g in rapeseed oil, 8.7 µg/g in sunflower oil). The highest level of CoQ10 in green tobacco leaves (27.6 µg/g) was found by Stiff et al. [18]; in broccoli (11.3 µg/g), tomato (2.6 µg/g), carrot (4.8 µg/g), pea (3.3 µg/g), orange (3.9 µg/g), kiwi (2.6 µg/g), apricot (4.6 µg/g), and cherry (14.5 µg/g) by Li et al. [28]; in cauliflower (6.6 µg/g), in potato (1.6 µg/g) and in bean (2.3 µg/g) by Kubo et al. [30]; in parsley (11.4 µg/g), spinach (13.5 µg/g), avocado (24.3 µg/g), and peanut (25.5 µg/g) by Román-Pizarro et al. [21]; in apple (1.3 µg/g), strawberry (1.4 µg/g), and rapeseed oil (63.5 µg/g) by Mattila and Kumpulainen [24]; in sesame oil (31.5 µg/g) by Pyo [7]; and in soybean oil (97.6 µg/g) by Rodríguez-Acuña et al. [27].

Regarding products of animal origin (Table 2), the contents of CoQ10 were investigated in meat and poultry (10.6–192.0 µg/g; 157.9 µg/g for reindeer meat, 13.6–45.1 µg/g for pork meat, 19.2–128.7 µg/g for pork heart, 21.1–53.6 µg/g for pork liver, 18.3–96.4 µg/g for pork kidney, 35.1 µg/g for pork brain, 16.1–48.8 µg/g for beef meat, 60.5–113.3 µg/g for beef heart, 33.3–50.5 µg/g for beef liver, 14.7 µg/g for lamb meat, 10.6–21.1 µg/g for chicken meat, 192.0 µg/g for chicken heart), fish and seafood (1.7–134.2 µg/g; 10.6–25.8 µg/g for mackerel flesh, 13.0 µg/g for horse mackerel flesh, 19.4 µg/g for herring flesh, 11.2–13.9 µg/g for Baltic herring flesh, 11.9–30.5 µg/g for sardine flesh, 25.4–27.5 µg/g for saury flesh, 12.8 µg/g for five-ray yellowtail flesh, 33.4 µg/g for young yellowtail flesh, 3.7 µg/g for cod flesh, 5.7 µg/g for salmon flesh, 4.9 µg/g for tuna flesh, 1.8 µg/g for flatfish flesh, 14.4 µg/g for pollack flesh, 18.6 µg/g for whole mackerel, 9.9µg/g for whole herring, 107.7 µg/g for mackerel heart, 134.2 µg/g for herring heart, 5.0 µg/g for scallop flesh, 3.4 µg/g for oyster flesh, 4.7 µg/g for cuttlefish flesh, 3.4 µg/g for octopus flesh, 1.7 µg/g for shrimp flesh), fish oils (70.9–286.1 µg/g; 70.9–133.3 µg/g for mackerel oil, 150.9–286.1 µg/g for herring oil), eggs (0.73–1.5 µg/g), milk and dairy (0.10–2.4 µg/g; 0.10 µg/g for skimmed milk, 0.30–0.31 µg/g for whole milk, 0.26–2.4 µg/g for yoghurt, 1.4 µg/g for cheese, 1.3 µg/g for Emmental cheese, 1.2 µg/g for Edam cheese, 0.16 µg/g for hard cheese, 0.29 µg/g for cream cheese, 1.6 µg/g for Provola cheese, 1.4 µg/g for Pecorino cheese, 1.3 µg/g for Bagoss cheese), and bee pollen (3.7–87.2 µg/g). The highest levels of CoQ10 in pork meat (45.1 µg/g) and sardine flesh (30.5 µg/g) were obtained by Li et al. [28], in pork heart (128.7 µg/g), liver (53.6 µg/g), and kidney (96.4 µg/g) by Niklowitz et al. [3], in beef meat (48.8 µg/g) by Tobin et al. [35], in beef heart (113.3 µg/g) and yoghurt (2.4 µg/g) by Mattila and Kumpulainen [24], in beef liver (50.5 µg/g), chicken meat (21.1 µg/g), and milk (0.31 µg/g) by Kubo et al. [30], in mackerel flesh (25.8 µg/g) by Souchet and Laplante [17], and in hen’s egg (1.5 µg/g) by Weber et al. [16].

### 3.1. Advantages and Disadvantages of Analytical Methods

This subsection highlights the advantages and disadvantages of analytical methods that best quantify CoQ10 from food products (Table S1). For example, the direct extraction method with 2-propanol proposed by Stiff et al. [18] for recovering CoQ10 from tobacco leaves has significant advantages (involves simple extraction steps, a single extraction is sufficient, and no additional purification is necessary). On the other hand, the HPLC method of Stiff et al. [18] is rapid, robust, and reproducible. In addition, it resulted in the excellent resolution of CoQ10 from other lipophilic components of the 2-propanol extract.

One benefit of the direct extraction method used by Li et al. [28] consists of its using a single extractant containing a mixture of ethanol/water (95:5, *v*/*v*); this protic solution increases the amperometric response of CoQ10 analysis compared to an aprotic solvent or a weak protic solvent such as 2-propanol [56]. In addition, it is environmentally friendly compared to previous methods [38,57,58,59]. Yet, the procedure is laborious, time-consuming, and requires three extraction processes; moreover, incubation in boiling water of the collective extract in order to concentrate the analyte under nitrogen could cause heat denaturation of CoQ10, leading thus to the underestimation of its level. Additionally, the DPV technique used in this study is feasible for determining CoQ10 in complex samples, demonstrating high precision and accuracy. Furthermore, it facilitates the highly selective and sensitive detection of CoQ10.

To protect ubiquinol-10 from oxidation during sample preparation, Kubo et al. [30] used a simple and fast isolation procedure that consists of homogenization in 2-propanol and centrifugation to separate the supernatant for HPLC injection, with no concentration or solvent substitution steps. The drawback of this extraction method is that it cannot concentrate analytes of ubiquinol-10 and ubiquinone-10, which exist at low concentrations in foods. Nevertheless, the combination of a reduction column and amperometric EC detector employed by Kubo et al. [30], chosen due to its easy maintenance, has proven to be a sensitive and reliable tool for the simultaneous analysis of ubiquinol-10 and ubiquinone-10 in a food matrix with HPLC.

The direct ethanol/*n*-hexane extraction method implemented by Román-Pizarro et al. [21] has been previously reported by Weber et al. [16], Mattila et al. [31], Mattila and Kumpulainen [24], Purchas et al. [22], Souchet and Laplante [17], Laplante et al. [48], Ercan and El [5], and Tobin et al. [35] with some differences. Among the benefits of this direct extraction method are that it is simple to perform and ensures the conversion of all coenzyme Q into the oxidized form [24]. In addition, according to Weber et al. [16], it gives comparable results to the saponification procedure. However, the protocol used by Román-Pizarro et al. [21] necessitates three extraction processes, uses more reagents than the direct extraction method with 2-propanol, and is not suitable for some food products [24]. As for the fluorescence spectroscopy method applied by them [21] for quantifying CoQ10 using a Cary Eclipse spectrofluorimeter (Varian), this is distinguished by improved sensitivity, good selectivity, the lack of potential interferences from the sample matrix, and practical utility. In addition, the required equipment is simpler and more cost-effective than liquid chromatography instruments.

Both UV and EC detection can be employed in coenzyme Q analysis. However, although EC detection is much more sensitive than DA detection, it lacks selectivity in some cases [31]. Hence, in their subsequent study, Mattila and Kumpulainen [24] quantified coenzymes Q9 and Q10 using DA detection. The chromatographic conditions used here effectively separated coenzymes Q9 and Q10 from each other and the matrix, allowing the reliable quantification of these compounds.

Pyo [7] accurately quantified CoQ9 and CoQ10 in rapeseed and sesame oil using saponification before solvent extraction, according to the method described by Mattila and Kumpulainen [24], for food samples such as pork liver, beef liver, rapeseed oil, and Emmental cheese. Although efficient, this complex extraction procedure is laborious, time-consuming, and uses many reagents; therefore, it is more cost-intensive. As for the HPLC/ESI-MS method used, the researcher chose it for molecular characterization and analytical purposes due to its high sensitivity and selectivity [60]. Nonetheless, the simultaneous determination of CoQ9 and CoQ10 in food items using LC-MS is rare.

In the extraction procedure developed by Rodríguez-Acuña et al. [27], the SPE cleanup step applied to remove matrix interferences allows minimum sample manipulation (one-step sample cleanup), as well as having lower solvent and time requirements, than direct ethanol/*n*-hexane extraction [31] or extraction preceded by saponification [24]. Therefore, compared to the previous methods [24,31], this procedure is environmentally friendly and increases the throughput of CoQ analysis. Furthermore, the detection technique, based on the highly efficient formation of its ions by APCI(-) and the minimization of interferences by using the selective reaction monitoring mode, increases the sensitivity and selectivity of the method. Besides this, the low amount of sample needed (because of the method’s high sensitivity) and the high purity of extract (resulting from the SPE cleanup) increase the HPLC column’s lifetime.

The use of cold 2-propanol for swine tissue homogenization followed by hexane extraction proposed by Niklowitz et al. [3] is an effective extraction method for the lipophilic CoQ10 that offers good precision and analytical recovery, with a demonstrated stability of concentration and redox status of at least three months at −84 °C. Furthermore, the HPLC method used in this study is sensitive and reproducible for the simultaneous measurement of oxidized and reduced CoQ10 forms in the tissue homogenate. For hexane extraction, the propanol homogenate can be used directly, or if a protein analysis of the pellet is intended, its supernatant resulting after centrifugation can be utilized. The separated hexane phase is evaporated to dryness and redissolved in alcohol before injection into the HPLC system; even if this procedure is more time-consuming than direct injection into the HPLC instrument of filtered propanol homogenate [61], it increases the purity and concentration of the injected sample.

Since Tobin et al. [35] used the methods described by Mattila and Kumpulainen [24] for the extraction and quantification of CoQ10 in beef meat, their advantages, as well as their disadvantages, which are mentioned in the lines above, also apply here.

The extraction method proposed by Souchet and Laplante [17] to determine CoQ10 content in fish flesh, based on a modification of the procedure previously reported by Lang et al. [62], Weber et al. [16], and Mattila and Kumpulainen [24], has the benefits and drawbacks mentioned in the fourth line of the current subsection. As for quantification, a valuable, fast, and simple HPLC-DAD technique was used; the optimized composition of the mobile phase allows a rapid elution of the sample, thus saving time and solvents significantly. Furthermore, it shows excellent sensitivity, reproducibility, and recovery, as well as good accuracy, since there is no matrix effect. In addition, no post-run column washout is required before the next injection; therefore, many samples (>100) can be run without post-run time or column cleaning.

The advantages and disadvantages of the direct ethanol/*n*-hexane extraction method used by Weber et al. [16] to extract CoQ10 from hen’s eggs are mentioned in the fourth paragraph of this subsection. Furthermore, regarding the HPLC method used by them for quantification, the better selectivity of DA detection compared to the EC approach [31] used by Kubo et al. [30] could be the reason for the higher level of CoQ10 found in hen’s eggs.

## 4. Conclusions

The analytical methods available in the literature for determining CoQ10 contents in foods consist of a combination of extraction and quantification techniques, each with strengths and limitations. The extraction process is critical and must be chosen based on matrix complexity. Generally, a direct extraction method is used with 2-propanol or a mixture of ethanol/*n*-hexane, and sometimes ethanol/water; an ultrasound-assisted sample pretreatment may be applied to improve the extraction yield. For complex matrices, a saponification step is needed before extraction to remove the interferences, or a purification step afterwards; in these cases, the saponification extraction method and solid-phase extraction (SPE), respectively, are used. Accelerated solvent extraction (ASE) may also be applied; this provides greater extraction efficiency with low solvent volumes and a shorter extraction time than the others. High-performance liquid chromatography, spectrofluorimetry, and differential pulse voltammetry are used to analyze CoQ10 quantitatively in the obtained extracts. The richest sources of CoQ10 are oils, organs, and meat.

## Figures and Tables

**Table 1:** Contents of CoQ10 in products of vegetable origin.

Food Source	Extraction Method	Detection Method and Quantification Method	CoQ10 Content	Limit of Quantification (LOQ)	Limit of Detection (LOD)	Linear Range	Spike Concentration	Recovery Rate	Ref.
Herbs									
Tobacco-green leaf	Ultrasonic extraction method with anhydrous ethanol and hexane	HPLC/ESI-MS/MS detectionSA quantification WSR: 8.4–540 ng/mL	11.5 µg/g	4.0 ng/mL	1.2 ng/mL	8.4–540.0 ng/mL	50 ng	98.2%	[46]
							175 ng	99.3%	
							300 ng	98.6%	
	Direct extraction method with 2-propanol	HPLC with UV detection (275 nm) ES quantification WSR: unspecified	27.6 µg/g	-	0.063 µg/mL	0.158–10.14 µg/mL	-	-	[18]
Parsley	Direct extraction method with 2-propanol	HPLC with AEC detection (600 mV) ES quantification WSR: unspecified	7.5 µg/g	-	38 pg/injection corresponding to 0.07 µg/g for ubiquinol-10	0.040–50 ng/injection corresponding to 0.08–100 µg/g for ubiquinol-10	-	-	[30]
					38 pg/injection corresponding to 0.15 µg/g for ubiquinone-10	0.040–50 ng/injection corresponding to 0.16–200 µg/g for ubiquinone-10			
	Direct extraction method with 0.15 M sodium chloride solution, ethanol, *n*-hexane, and acetone	A. CEFS detection (585 and 627 nm) ES quantification WSR: 35.0–500 nmol/L	11.4 µg/g	-	0.008 µmol/L	0.03–0.50 µmol/L	4.3 µg/g	88.9%	[21]
							8.6 µg/g	88.0%	
		B. HPLC with DA detection (275 nm) ES quantification WSR: 2.0–200 µmol/L	11.1 µg/g	-	0.58 µmol/L	-	-	-	
Perilla	Direct extraction method See parsley	See parsley	2.1 µg/g	-	See parsley	See parsley	-	-	[30]
Rape-leaf	Direct extraction method See parsley	See parsley	6.7 µg/g	-	See parsley	See parsley	-	-	[30]
Vegetables									
Broccoli	Direct extraction method See parsley	See parsley	7.0 µg/g	-	See parsley	See parsley	-	-	[30]
	Direct extraction method with nitrogen-saturated ethanol/water (95:5, *v*/*v*) PBS solution (pH 6.5)	A. DPV using an electrochemical workstation CV scanning range: −0.10 V to −0.80 V DPV initial potential: −0.01 V DPV final potential: −0.80 V DPV amplitude: 0.05 V	11.3 µg/g	-	0.0288 mg/kg (3.3 × 10^−8^ mol/L)	0.0863–863 mg/kg (1.00 × 10^−7^–1.00 × 10^−3^ mol/L)	5.0 mg/kg	91.0–108.0%	[28]
		B. HPLC with UV detection (274 nm) Quantification method: unspecified	10.5 µg/g	-	-	-	-	-	
Cauliflower	Direct extraction method with 0.15 M sodium chloride solution, ethanol, *n*-hexane, 2-propanol	HPLC with DA detection (275 nm) ES quantification WSR: 2.5–55 µg/mL	2.7 µg/g	-	5 ng/injection	12–500 ng/injection	Unspecified	93.0%	[24]
	Direct extraction method See parsley	See parsley	6.6 µg/g	-	See parsley	See parsley	-	-	[30]
Cabbage	Direct extraction method See parsley	See parsley	3.8 µg/g	-	See parsley	See parsley	-	-	[30]
Potato	Direct extraction method See cauliflower	See cauliflower	0.50 µg/g	-	See cauliflower	See cauliflower	See cauliflower	See cauliflower	[24]
	Direct extraction method See parsley	See parsley	1.6 µg/g	-	See parsley	See parsley	0.44 µg/g ubiquinol-10	112.0%	[30]
							4.00 µg/g ubiquinol-10	104.0%	
							0.22 µg/g ubiquinone-10	101.0%	
							1.96 µg/g ubiquinone-10	98.2%	
Tomato	Direct extraction method with 0.9% sodium chloride solution, ethanol/hexane (1:5, *v*/*v*), sodium sulfate anhydrous, hexane	HPLC with UV detection (275 nm) ES quantification WSR: unspecified	0.19 µg/g	-	-	-	-	-	[16]
	Direct extraction method See cauliflower	See cauliflower	0.90 µg/g	-	See cauliflower	See cauliflower	See cauliflower	See cauliflower	[24]
	Direct extraction method See broccoli	See broccoli A	2.6 µg/g	-	See broccoli	See broccoli	-	-	[28]
		See broccoli B	2.2 µg/g	-	-	-	-	-	
Carrot	Direct extraction method See tomato	See tomato	<0.24 µg/g	-	-	-	-	-	[16]
	Direct extraction method See cauliflower	See cauliflower	1.7 µg/g	-	See cauliflower	See cauliflower	See cauliflower	See cauliflower	[24]
	Direct extraction method See broccoli	See broccoli A	4.8 µg/g	-	See broccoli	See broccoli	-	-	[28]
		See broccoli B	3.6 µg/g	-	-	-	-	-	
Cucumber	Direct extraction method See tomato	See tomato	<0.08 µg/g	-	-	-	-	-	[16]
	Direct extraction method See parsley	See parsley	0.08 µg/g	-	See parsley	See parsley	-	-	[30]
Corn	Direct extraction method See broccoli	See broccoli A	5.1 µg/g	-	See broccoli	See broccoli	5.0 mg/kg	105.4%	[28]
		See broccoli B	4.4 µg/g	-	-	-	-	-	
Spinach	Direct extraction method See parsley	See parsley	0.44 µg/g	-	See parsley	See parsley	-	-	[30]
	Direct extraction method See parsley	See parsley A	13.5 µg/g	-	See parsley A	See parsley A	4.3 µg/g	93.6%	[21]
							8.6 µg/g	95.9%	
		See parsley B	12.5 µg/g	-	See parsley B	-	-	-	
	Direct extraction method See broccoli	See broccoli A	7.2 µg/g	-	See broccoli	See broccoli	-	-	[28]
		See broccoli B	6.7 µg/g	-	-	-	-	-	
Mustard spinach	Direct extraction method See parsley	See parsley	2.0 µg/g	-	See parsley	See parsley	-	-	[30]
Eggplant	Direct extraction method See parsley	See parsley	1.0 µg/g	-	See parsley	See parsley	-	-	[30]
Radish	Direct extraction method See parsley	See parsley	0.70 µg/g	-	See parsley	See parsley	-	-	[30]
Onion	Direct extraction method See parsley	See parsley	0.90 µg/g	-	See parsley	See parsley	-	-	[30]
Garlic	Direct extraction method See parsley	See parsley	3.5 µg/g	-	See parsley	See parsley	-	-	[30]
Lotus root	Direct extraction method See parsley	See parsley	0.96 µg/g	-	See parsley	See parsley	-	-	[30]
Pea	Direct extraction method See cauliflower	See cauliflower	2.7 µg/g	-	See cauliflower	See cauliflower	See cauliflower	See cauliflower	[24]
	Direct extraction method See parsley	See parsley	2.3 µg/g	-	See parsley	See parsley	-	-	[30]
	Direct extraction method See broccoli	See broccoli A	3.3 µg/g	-	See broccoli	See broccoli	-	-	[28]
		See broccoli B	2.5 µg/g	-	-	-	-	-	
Bean	Direct extraction method See cauliflower	See cauliflower	1.8 µg/g	-	See cauliflower	See cauliflower	See cauliflower	See cauliflower	[24]
	Direct extraction method See parsley	See parsley	2.3 µg/g	-	See parsley	See parsley	-	-	[30]
Soybean	Direct extraction method See parsley	See parsley	6.8 µg/g	-	See parsley	See parsley	-	-	[30]
Asparagus	Direct extraction method See parsley	See parsley	2.2 µg/g	-	See parsley	See parsley	-	-	[30]
Avocado	Direct extraction method See parsley	See parsley	9.5 µg/g	-	See parsley	See parsley	-	-	[30]
	Direct extraction method See parsley	See parsley A	24.3 µg/g	-	See parsley A	See parsley A	4.3 µg/g	88.2%	[21]
							8.6 µg/g	90.3%	
		See parsley B	13.2 µg/g	-	See parsley B	-	-	-	
Fruits									
Orange	Direct extraction method See tomato	See tomato	2.2 µg/g	-	-	-	-	-	[16]
	Direct extraction method See cauliflower	See cauliflower	1.4 µg/g	-	See cauliflower	See cauliflower	See cauliflower	See cauliflower	[24]
	Direct extraction method See parsley	See parsley	1.0 µg/g	-	See parsley	See parsley	-	-	[30]
	Direct extraction method See broccoli	See broccoli A	3.9 µg/g	-	See broccoli	See broccoli	-	-	[28]
		See broccoli B	3.3 µg/g	-	-	-	-	-	
Clementine	Direct extraction method See cauliflower	See cauliflower	0.90 µg/g	-	See cauliflower	See cauliflower	See cauliflower	See cauliflower	[24]
Apple	Direct extraction method See tomato	See tomato	1.1 µg/g	-	-	-	-	-	[16]
	Direct extraction method See cauliflower	See cauliflower	1.3 µg/g	-	See cauliflower	See cauliflower	See cauliflower	See cauliflower	[24]
	Direct extraction method See parsley	See parsley	1.2 µg/g	-	See parsley	See parsley	-	-	[30]
Blackcurrant	Direct extraction method See cauliflower	See cauliflower	3.4 µg/g	-	See cauliflower	See cauliflower	See cauliflower	See cauliflower	[24]
Lingonberry	Direct extraction method See cauliflower	See cauliflower	0.90 µg/g	-	See cauliflower	See cauliflower	See cauliflower	See cauliflower	[24]
Strawberry	Direct extraction method See cauliflower	See cauliflower	1.4 µg/g	-	See cauliflower	See cauliflower	See cauliflower	See cauliflower	[24]
	Direct extraction method See parsley	See parsley	0.50 µg/g	-	See parsley	See parsley	-	-	[30]
Grapefruit	Direct extraction method See parsley	See parsley	1.3 µg/g	-	See parsley	See parsley	-	-	[30]
Banana	Direct extraction method See parsley	See parsley	0.80 µg/g	-	See parsley	See parsley	-	-	[30]
Kiwi	Direct extraction method See tomato	See tomato	0.49 µg/g	-	-	-	-	-	[16]
	Direct extraction method See broccoli	See broccoli A	2.1 µg/g	-	See broccoli	See broccoli	1.0 mg/kg	91.4%	[28]
		See broccoli B	2.6 µg/g	-	-	-	-	-	
Persimmon	Direct extraction method See parsley	See parsley	0.80 µg/g	-	See parsley	See parsley	-	-	[30]
Apricot	Direct extraction method See broccoli	See broccoli A	4.1 µg/g	-	See broccoli	See broccoli	-	-	[28]
		See broccoli B	4.6 µg/g	-	-	-	-	-	
Cherry	Direct extraction method See broccoli	See broccoli A	12.2 µg/g	-	See broccoli	See broccoli	-	-	[28]
		See broccoli B	14.5 µg/g	-	-	-	-	-	
Dry date	Saponification extraction method Saponification with water, pyrogallol, methanol, 25% aqueous potassium hydroxide solution, petroleum ether 40–60 °C, sodium sulfate anhydrous, ethanol Purification of the saponified extract over alumina column Separation of CoQ10 from the purified extract on silica gel F_254_ glass plate	HPLC with UV detection (275 nm) Quantification method: unspecified	21.1 µg/g	-	-	-	-	-	[47]
Grains & seeds									
Almond	Direct extraction method See parsley	See parsley	5.0 µg/g	-	See parsley	See parsley	-	-	[30]
Peanut	Direct extraction method See parsley	See parsley A	20.9 µg/g	-	See parsley A	See parsley A	4.3 µg/g	89.3%	[21]
							8.6 µg/g	101.3%	
		See parsley B	25.5 µg/g	-	See parsley B	-	-	-	
	Direct extraction method See broccoli	See broccoli A	11.5 µg/g	-	See broccoli	See broccoli	10.0 mg/kg	91.8%	[28]
		See broccoli B	12.6 µg/g	-	-	-	-	-	
Pistachio	Direct extraction method See parsley	See parsley A	18.5 µg/g	-	See parsley A	See parsley A	4.3 µg/g	91.3%	[21]
							8.6 µg/g	84.5%	
		See parsley B	22.2 µg/g	-	See parsley B	-	-	-	
Rapeseed	Direct extraction method See broccoli	See broccoli A	3.2 µg/g	-	See broccoli	See broccoli	-	-	[28]
		See broccoli B	3.0 µg/g	-	-	-	-	-	
Barley	Direct extraction method See broccoli	See broccoli A	9.7 µg/g	-	See broccoli	See broccoli	-	-	[28]
		See broccoli B	8.2 µg/g	-	-	-	-	-	
Vegetable oils									
Olive oil	Saponification extraction method with 5% aqueous pyrogallol solution, 10% sodium hydroxide solution, methanol, 10% sodium chloride solution, *n*-hexane, 5% sodium chloride solution, ethanol, 2-propanol	HPLC/ESI-MS detection Quantification method: unspecified	1.3 µg/g	-	-	-	-	-	[7]
Sesame oil	Saponification extraction method See olive oil	See olive oil	31.5 µg/g	-	-	-	-	-	[7]
	Direct extraction method See parsley	See parsley	17.6 µg/g	-	See parsley	See parsley	-	-	[30]
Maize germ oil	Saponification extraction method See olive oil	See olive oil	17.7 µg/g	-	-	-	-	-	[7]
Perilla oil	Saponification extraction method See olive oil	See olive oil	84.9 µg/g	-	-	-	-	-	[7]
Grape seed oil	Saponification extraction method See olive oil	See olive oil	20.2 µg/g	-	-	-	-	-	[7]
Soybean oil	Saponification extraction method See olive oil	See olive oil	54.2 µg/g	-	-	-	-	-	[7]
	Direct extraction method See parsley	See parsley	53.8 µg/g	-	See parsley	See parsley	-	-	[30]
	Solid-phase extraction (SPE) with heptane, heptane/ethyl ether (80:20, *v*/*v*), acetonitrile/tetrahydrofuran (90:10, *v*/*v*) 5 g of solid-phase extraction (SPE) cartridge with amino-propyl (NH_2_) adsorbents, Varian	HPLC/APCI-MS detection SA quantification Working standards: 51.1 mg/kg CoQ10 and 105.4 mg/kg CoQ10	97.6 µg/g	60 pg/injection corresponding to 0.025 mg/kg oil	18 pg/injection	-	-	-	[27]
Rapeseed oil	Saponification extraction method with 2% ascorbic acid solution, methanol, aqueous potassium hydroxide solution (50 g KOH + 50 mL H_2_O), 10% sodium chloride solution, *n*-hexane, 5% sodium chloride solution, ethanol, *n*-hexane/2-propanol (1:1, *v*/*v*)	See cauliflower	63.5 µg/g	-	See cauliflower	See cauliflower	See cauliflower	See cauliflower	[24]
	Solid-phase extraction (SPE) See soybean oil	See soybean oil Working standards: 26.2 mg/kg CoQ10 and 51.5 mg/kg CoQ10	46.4 µg/g	See soybean oil	See soybean oil	-	-	-	[27]
Sunflower oil	Solid-phase extraction (SPE) See soybean oil	See soybean oil Working standards: 10.5 mg/kg CoQ10 and 15.9 mg/kg CoQ10	8.7 µg/g	See soybean oil	See soybean oil	-	-	-	[27]

HPLC—high-performance liquid chromatography; UV—ultraviolet; ES—external standard; WSR—working standards range; HPLC/ESI-MS/MS—high-performance liquid chromatography–electrospray ionization mass spectrometry–tandem mass spectrometry; SA—standard addition; AEC—amperometric electrochemical; CEFS—Cary Eclipse fluorescence spectrometer; DA—diode-array; DPV—differential pulse voltammetry; CV—cyclic voltammetry; HPLC/APCI-MS—high-performance liquid chromatography–atmospheric pressure chemical ionization mass spectrometry; Ref.—reference.

**Table 2:** Contents of CoQ10 in products of animal origin.

Food Source	Extraction Method	Detection Method and Quantification Method	CoQ10 Content	Limit of Quantification (LOQ)	Limit of Detection (LOD)	Linear Range	Spike Concentration	Recovery Rate	Ref.
Meat and Poultry									
Reindeer meat	Direct extraction method See cauliflower	See cauliflower	157.9 µg/g	-	See cauliflower	See cauliflower	See cauliflower	See cauliflower	[24]
Pork meat	Direct extraction method with Hanks′ balanced salt solution, ethanol, *n*-hexane, 2-propanol	HPLC with UV detection (275 nm) ES quantification Working standards range: 2.0–200 µg/mL	41.6 µg/g	-	-	-	1 mg/g	Unspecified	[35]
	Direct extraction method See broccoli	See broccoli A	45.1 µg/g	-	See broccoli	See broccoli	-	-	[28]
		See broccoli B	13.6 µg/g	-	-	-	-	-	
	Direct extraction method See parsley	See parsley	29.4 µg/g	-	See parsley	See parsley	-	-	[30]
	Direct extraction method with 2-propanol, saline solution, hexane, methanol/ethanol/propanol (100:95:5, *v*/*v*/*v*)	HPLC with EC detection IS quantification Working standards: 310 pmol ubihydroquinone-9 and 400 pmol ubiquinone-9 in 50 µL ethanol	23.1 µg/g	-	-	12–60 mg fresh muscle tissue/sample	192.4 pm/sample ubiquinone-10	86.3%	[3]
							105.3 pm/sample ubiquinone-10	96.8%	
							51.4 pm/sample ubiquinone-10	88.2%	
							136.6 pm/sample ubihydroquinone-10	98.6%	
							75.9 pm/sample ubihydroquinone-10	101.4%	
							33.3 pm/sample ubihydroquinone-10	110.5%	
							328.9 pm/sample total CoQ10	90.0%	
							181.2 pm/sample total CoQ10	98.1%	
							84.7 pm/sample total CoQ10	93.9%	
Pork heart	Direct extraction method with 0.15 M sodium chloride solution, ethanol, *n*-hexane, sodium sulfate anhydrous, 2-propanol	A. HPLC with DA detection (275 nm) ES quantification Working standards range: unspecified	63.4 µg/g	-	6 ng/injection	10–200 ng/injection	18–60 µg	73.0–105.0%	[31]
		B. HPLC with CMEA	63.5 µg/g	-	0.3 ng/injection	10–200 ng/injection	18–60 µg	74.0–103.0%	
	Direct extraction method See cauliflower	See cauliflower	126.8 µg/g	-	See cauliflower	See cauliflower	See cauliflower	See cauliflower	[24]
	Direct extraction method See broccoli	See broccoli A	19.2 µg/g	-	See broccoli	See broccoli	-	-	[28]
		See broccoli B	20.5 µg/g	-	-	-	-	-	
	Direct extraction method See pork meat	See pork meat	128.7 µg/g	-	-	See pork meat	See pork meat	See pork meat	[3]
Pork liver	Saponification extraction method with 2% ascorbic acid solution, methanol, potassium hydroxide solution (50 g KOH + 50 mL H_2_O), 10% sodium chloride solution, *n*-hexane, 5% sodium chloride solution, ethanol, *n*-hexane/2-propanol (3:7, *v*/*v*)	See cauliflower	22.7 µg/g	-	See cauliflower	See cauliflower	See cauliflower	See cauliflower	[24]
	Direct extraction method See parsley	See parsley A	45.1 µg/g	-	See parsley A	See parsley A	4.3 µg/g	97.0%	[21]
							8.6 µg/g	87.9%	
		See parsley B	45.7 µg/g	-	See parsley B	-	-	-	
	Direct extraction method See broccoli	See broccoli A	21.1 µg/g	-	See broccoli	See broccoli	-	-	[28]
		See broccoli B	22.2 µg/g	-	-	-	-	-	
	Direct extraction method See pork meat	See pork meat	53.6 µg/g	-	-	See pork meat	See pork meat	See pork meat	[3]
Pork kidney	Direct extraction method See broccoli	See broccoli A	18.3 µg/g	-	See broccoli	See broccoli	-	-	[28]
		See broccoli B	23.2 µg/g	-	-	-	-	-	
	Direct extraction method See pork meat	See pork meat	96.4 µg/g	-	-	See pork meat	See pork meat	See pork meat	[3]
Pork brain	Direct extraction method See pork meat	See pork meat	35.1 µg/g	-	-	See pork meat	See pork meat	See pork meat	[3]
Beef meat	Direct extraction method See pork meat	See pork meat	48.8 µg/g	-	-	-	See pork meat	See pork meat	[35]
	Direct extraction method See broccoli	See broccoli A	16.3 µg/g	-	See broccoli	See broccoli	10.0 mg/kg	108.3%	[28]
		See broccoli B	19.3 µg/g	-	-	-	-	-	
	Direct extraction method with 0.15 M sodium chloride solution, ethanol, *n*-hexane, 2-propanol Lyophilized sample	HPLC with UV detection (275 nm) Quantification method: unspecified	44.9 µg/g	-	-	-	-	-	[22]
	Direct extraction method See cauliflower	See cauliflower	36.5 µg/g	-	See cauliflower	See cauliflower	See cauliflower	See cauliflower	[24]
	Direct extraction method with 0.15 M sodium chloride solution, ethanol, *n*-hexane, 2-propanol	HPLC with UV detection (275 nm) ES quantification WSR: 2.5–55 µg/mL	23.5 µg/g	-	-	-	-	-	[5]
	Direct extraction method See parsley	See parsley	35.2 µg/g	-	See parsley	See parsley	2.0 µg/g ubiquinol-10	94.7%	[30]
							18.0 µg/g ubiquinol-10	87.8%	
							8.8 µg/g ubiquinone-10	97.4%	
							80.0 µg/g ubiquinone-10	101.0%	
	Direct extraction method See pork heart	See pork heart A	17.3 µg/g	-	See pork heart	See pork heart	See pork heart	See pork heart	[31]
		See pork heart B	16.1 µg/g	-	See pork heart	See pork heart	See pork heart	See pork heart	
Beef heart	Direct extraction method See cauliflower	See cauliflower	113.3 µg/g	-	See cauliflower	See cauliflower	See cauliflower	See cauliflower	[24]
	Direct extraction method See beef meat Lyophilized sample	See beef meat	60.5 µg/g	-	-	-	-	-	[22]
	Direct extraction method See beef meat	See beef meat	110.0 µg/g	-	-	-	-	-	[5]
Beef liver	Saponification extraction method See pork liver	See cauliflower	39.2 µg/g	-	See cauliflower	See cauliflower	See cauliflower	See cauliflower	[24]
	Direct extraction method See beef meat Lyophilized sample	See beef meat	46.0 µg/g	-	-	-	-	-	[22]
	Direct extraction method See parsley	See parsley	50.5 µg/g	-	See parsley	See parsley	-	-	[30]
	Saponification extraction method with 2% ascorbic acid solution, methanol, potassium hydroxide solution (50 g KOH + 50 mL H_2_O), 10% sodium chlorine solution, *n*-hexane, 5% sodium chloride solution, ethanol, *n*-hexane/2-propanol (30:70, *v*/*v*)	See beef meat	33.3 µg/g	-	-	-	-	-	[5]
	Direct extraction method See parsley	See parsley A	47.2 µg/g	-	See parsley A	See parsley A	4.3 µg/g	90.2%	[21]
							8.6 µg/g	83.5%	
		See parsley B	44.1 µg/g	-	See parsley B	-	-	-	
Lamb meat	Direct extraction method See beef meat Lyophilized sample	See beef meat	14.7 µg/g	-	-	-	-	-	[22]
Chicken meat	Direct extraction method See cauliflower	See cauliflower	14.0 µg/g	-	See cauliflower	See cauliflower	See cauliflower	See cauliflower	[24]
	Direct extraction method See broccoli	See broccoli A	10.6 µg/g	-	See broccoli	See broccoli	-	-	[28]
		See broccoli B	12.3 µg/g	-	-	-	-	-	
	Direct extraction method See parsley	See parsley	21.1 µg/g	-	See parsley	See parsley	-	-	[30]
Chicken heart	Direct extraction method See parsley	See parsley	192.0 µg/g	-	See parsley	See parsley	-	-	[30]
Fish & Seafood									
Mackerel flesh	Direct extraction method with 0.15 M sodium chloride solution, 0.1 M sodium dodecyl sulfate, anhydrous ethanol, hexane, 2-propanol	HPLC with DA detection (275 nm) ES quantification WSR: 2.5–55 µg/mL	25.8 µg/g	-	2.5 ng/injection	1–20 µg/mL corresponding to 10–200 µg/g fresh tissue	1–15 µg	105.1%	[17]
	Direct extraction method See parsley	See parsley	10.6 µg/g	-	See parsley	See parsley	-	-	[30]
Horse mackerel flesh	Direct extraction method See parsley	See parsley	13.0 µg/g	-	See parsley	See parsley	-	-	[30]
Herring flesh	Direct extraction method See mackerel flesh	See mackerel flesh	19.4 µg/g	-	See mackerel flesh	See mackerel flesh	-	-	[17]
Baltic herring flesh	Direct extraction method See pork heart	See pork heart A	11.2 µg/g	-	See pork heart	See pork heart	See pork heart	See pork heart	[31]
		See pork heart B	13.9 µg/g	-	See pork heart	See pork heart	See pork heart	See pork heart	
Sardine flesh	Direct extraction method See parsley	See parsley	11.9 µg/g	-	See parsley	See parsley	-	-	[30]
	Direct extraction method See broccoli	See broccoli A	30.5 µg/g	-	See broccoli	See broccoli	-	-	[28]
		See broccoli B	29.8 µg/g	-	-	-	-	-	
Saury flesh	Direct extraction method See broccoli	See broccoli A	25.4 µg/g	-	See broccoli	See broccoli	-	-	[28]
		See broccoli B	27.5 µg/g	-	-	-	-	-	
Five-ray yellowtail flesh	Direct extraction method See parsley	See parsley	12.8 µg/g	-	See parsley	See parsley	2.0 µg/g ubiquinol-10	105.0%	[30]
							18.0 µg/g ubiquinol-10	98.8%	
							3.0 µg/g ubiquinone-10	106.0%	
							26.8 µg/g uniquinonă-10	97.5%	
Young yellowtail flesh	Direct extraction method See parsley	See parsley	33.4 µg/g	-	See parsley	See parsley	-	-	[30]
Cod flesh	Direct extraction method See parsley	See parsley	3.7 µg/g	-	See parsley	See parsley	-	-	[30]
Salmon flesh	Direct extraction method See parsley	See parsley	5.7 µg/g	-	See parsley	See parsley	-	-	[30]
Tuna flesh	Direct extraction method See parsley	See parsley	4.9 µg/g	-	See parsley	See parsley	-	-	[30]
Flatfish flesh	Direct extraction method See parsley	See parsley	1.8 µg/g	-	See parsley	See parsley	-	-	[30]
Pollack flesh	Direct extraction method See cauliflower	See cauliflower	14.4 µg/g	-	See cauliflower	See cauliflower	See cauliflower	See cauliflower	[24]
Whole mackerel	Direct extraction method See Souchet and Laplante [17]	See Souchet and Laplante [17]	18.6 µg/g	-	-	-	-	-	[48]
	Direct extraction method See Souchet and Laplante [17] Lyophilized sample		88.4 µg/g dw	-	-	-	-	-	
Whole herring	Direct extraction method See Souchet and Laplante [17]	See Souchet and Laplante [17]	9.9 µg/g	-	-	-	-	-	[48]
	Direct extraction method See Souchet and Laplante [17] Lyophilized sample		50.9 µg/g dw	-	-	-	-	-	
Mackerel heart	Direct extraction method See mackerel flesh	See mackerel flesh	107.7 µg/g	-	See mackerel flesh	See mackerel flesh	-	-	[17]
Herring heart	Direct extraction method See mackerel flesh	See mackerel flesh	134.2 µg/g	-	See mackerel flesh	See mackerel flesh	1–15 µg	100.3%	[17]
Scallop flesh	Direct extraction method See parsley	See parsley	5.0 µg/g	-	See parsley	See parsley	-	-	[30]
Oyster flesh	Direct extraction method See parsley	See parsley	3.4 µg/g	-	See parsley	See parsley	-	-	[30]
Cuttlefish flesh	Direct extraction method See parsley	See parsley	4.7 µg/g	-	See parsley	See parsley	-	-	[30]
Octopus flesh	Direct extraction method See parsley	See parsley	3.4 µg/g	-	See parsley	See parsley	-	-	[30]
Shrimp flesh	Direct extraction method See parsley	See parsley	1.7 µg/g	-	See parsley	See parsley	-	-	[30]
Fish oils									
Mackerel oil	A. Oil extracted by enzymatic hydrolysis Direct extraction method with 2-propanol	See Souchet and Laplante [17]	133.3 µg/g	-	-	-	-	82.0%	[48]
	B. Oil extracted using SCO_2_ 600 g CO_2_/h + 5% EtOH Direct extraction method with 2-propanol		70.9 µg/g	-	-	-	-	33.0%	
Herring oil	A. Oil extracted by enzymatic hydrolysis Direct extraction method with 2-propanol	See Souchet and Laplante [17]	150.9 µg/g	-	-	-	-	84.0%	[48]
	B. Oil extracted using SCO_2_ 600 g CO_2_/h + 5% EtOH Direct extraction method with 2-propanol		286.1 µg/g	-	-	-	-	104.0%	
Eggs									
Hen’s egg	Direct extraction method See parsley	See parsley	0.73 µg/g	-	See parsley	See parsley	-	-	[30]
	Direct extraction method See tomato	See tomato	1.5 µg/g	-	-	-	-	-	[16]
	Direct extraction method See cauliflower	See cauliflower	1.2 µg/g	-	See cauliflower	See cauliflower	See cauliflower	See cauliflower	[24]
Milk & Dairy									
Skimmed milk (1.5% fat)	Direct extraction method with ethanol, *n*-hexane, 2-propanol	See cauliflower	0.10 µg/g	-	See cauliflower	See cauliflower	See cauliflower	See cauliflower	[24]
Whole milk	Direct extraction method See parsley	See parsley	0.31 µg/g	-	See parsley	See parsley	-	-	[30]
	Saponification extraction method with 2 N ethanolic potassium hydroxide solution, 1% ethanolic pyrogallol solution, bidistilled water, ethanol, petroleum ether/diethyl ether (9:1, *v*/*v*), sodium sulfate anhydrous, 2-propanol	HPLC with DA detection (275 nm) IS quantification Working standard: 15 µg ubiquinone-9	0.30 µg/g	1.18 µg/mL	0.35 µg/mL	-	-	-	[9]
Yoghurt	Direct extraction method See tomato	See tomato	1.2 µg/g	-	-	-	-	-	[16]
	Direct extraction method with ethanol, *n*-hexane, 2-propanol	See cauliflower	2.4 µg/g	-	See cauliflower	See cauliflower	See cauliflower	See cauliflower	[24]
	Direct extraction method See parsley	See parsley	0.26 µg/g	-	See parsley	See parsley	-	-	[30]
Cheese	Direct extraction method See parsley	See parsley	1.4 µg/g	-	See parsley	See parsley	-	-	[30]
Emmental cheese	Saponification extraction method See pork liver	See cauliflower	1.3 µg/g	-	See cauliflower	See cauliflower	See cauliflower	See cauliflower	[24]
Edam cheese	Saponification extraction method See pork liver	See cauliflower	1.2 µg/g	-	See cauliflower	See cauliflower	See cauliflower	See cauliflower	[24]
Hard cheese	Direct extraction method See tomato	See tomato	<0.16 µg/g	-	-	-	-	-	[16]
Cream cheese	Direct extraction method See tomato	See tomato	<0.29 µg/g	-	-	-	-	-	[16]
Provola cheese	Saponification extraction method with 60% potassium hydroxide solution, 6% ethanolic pyrogallol solution, 96% ethanol, 1% sodium chloride solution, hexane/ethyl acetate (9:1, *v*/*v*), 1% 2-propanol in *n*-hexane	HPLC with UV detection (275 nm) ES quantification WSR: 0.810–2.025 μg/mL	1.6 µg/g	0.069 µg/mL	0.024 µg/mL	0.810–2.025 μg/mL	2.025–0.810 µg/mL	98.6%	[39]
Pecorino cheese	Saponification extraction method See Provola cheese	See Provola cheese	1.4 µg/g	See Provola cheese	See Provola cheese	See Provola cheese	See Provola cheese	See Provola cheese	[39]
Bagoss cheese	Saponification extraction method See Provola cheese	See Provola cheese	1.3 µg/g	See Provola cheese	See Provola cheese	See Provola cheese	See Provola cheese	See Provola cheese	[39]
Bee products									
Rape bee pollen	Accelerated solvent extraction (ASE) with Cleanert Alumina-N, absolute ethanol Temperature: 80 °C Heat-up time: 5 min Static time: 5 min Flush volume: 60% Purge time: 1 min Number of cycles: 1 Cell volume: 10 mL Total extraction time: 16–17 min/sample	HPLC with DA detection (275 nm) ES quantification WSR: 0.25–200 mg/L	21.9 µg/g	0.35 mg/kg	0.16 mgk/g	0.25–200 mg/L	5 mg/L corresponding to 5 mg/kg sample	90.6%	[38]
							10 mg/L corresponding to 10 mg/kg sample	92.3%	
							50 mg/L corresponding to 50 mg/kg sample	95.1%	
Apricot bee pollen	Accelerated solvent extraction (ASE) See rape bee pollen	See rape bee pollen	87.2 µg/g	See rape bee pollen	See rape bee pollen	See rape bee pollen	See rape bee pollen	See rape bee pollen	[38]
Tea bee pollen	Accelerated solvent extraction (ASE) See rape bee pollen	See rape bee pollen	3.7 µg/g	See rape bee pollen	See rape bee pollen	See rape bee pollen	See rape bee pollen	See rape bee pollen	[38]
Mixed bee pollen	Accelerated solvent extraction (ASE) See rape bee pollen	See rape bee pollen	9.4 µg/g	See rape bee pollen	See rape bee pollen	See rape bee pollen	See rape bee pollen	See rape bee pollen	[38]

HPLC—high-performance liquid chromatography; UV—ultraviolet; ES—external standard; WSR—working standards range; DA—diode array; CMEA—coulometric multi-electrode electrochemical array; EC—electrochemical; IS—internal standard; dw—dry weight; Ref.—reference.

## Supplementary Materials

The following supporting information can be downloaded at: https://www.mdpi.com/article/10.3390/metabo13020272/s1, Table S1: Centralizing table of extraction methods used; Table S2: Centralizing table of quantification methods used; Figure S1: Workflow of the analytical method adapted from Zu et al., 2006; Figure S2: Workflow of the analytical method adapted from of Stiff et al., 2011; Figure S3: Workflow of the analytical method adapted from Kubo et al., 2008; Figure S4: Workflow of the analytical method adapted from Román-Pizarro et al., 2017; Figure S5: Workflow of the analytical method adapted from Li et al., 2016; Figure S6: Workflow of the analytical method adapted from Mattila and Kumpulainen, 2001 (direct extraction method); Figure S7: Workflow of the analytical method adapted from Mattila and Kumpulainen, 2001 (saponification-extraction method); Figure S8: Workflow of the analytical method adapted from Weber et al., 1997; Figure S9: Workflow of the analytical method adapted from Al-Faraji and Shanshal, 2010; Figure S10: Workflow of the analytical method adapted from Pyo, 2010; Figure S11: Workflow of the analytical method adapted from Rodríguez-Acuña et al., 2008; Figure S12: Workflow of the analytical method adapted from Tobin et al., 2014; Figure S13: Workflow of the analytical method adapted from Niklowitz et al., 2013; Figure S14: Workflow of the analytical method adapted from Mattila et al., 2000; Figure S15: Workflow of the analytical method adapted from Purchas et al., 2004; Figure S16: Workflow of the analytical method adapted from Souchet and Laplante, 2007; Figure S17: Workflow of the analytical method adapted from Mandrioli et al., 2018; Figure S18: Workflow of the analytical method adapted from Manzi and Durazzo, 2015; Figure S19: Workflow of the analytical method adapted from Xue et al., 2012.
