# Supplementary material for: An Overview of Analytical Methods for Quantitative Determination of Coenzyme Q10 in Foods
Source: Metabolites. 2023 Feb 14;13(2):272. doi: 10.3390/metabo13020272 (PMC9964353; doi:10.3390/metabo13020272)
Supplement: Supplementary file 1 [file metabolites-13-00272-s001.zip › metabolites-2207370-supplementary.pdf]

Supporting information for:

An Overview of Analytical Methods for Quantitative Determination of Coenzyme Q10 in Foods

Andersina Simina Podar [1,2], Cristina Anamaria Semeniuc [2,3,*], Simona Raluca Ionescu [2,3], Maria-Ioana Socaciu [2,3], Melinda Fogarasi [2,3], Anca Corina Fărcaş [1,2], Dan Cristian Vodnar [1,2] and Sonia Ancuța Socaci [1,2,*]

[1] Department of Food Science, University of Agricultural Sciences and Veterinary Medicine of Cluj-Napoca, 3-5 Mănăştur St., 400372 Cluj-Napoca, Romania; andersina-simina.podar@usamvcluj.ro (A.S.P.); anca.farcas@usamvcluj.ro (A.C.F.); dan.vodnar@usamvcluj.ro (D.C.V.)

[2] Centre for Technology Transfer-BioTech, 64 Calea Florești, 400509 Cluj-Napoca, Romania

[3] Department of Food Engineering, University of Agricultural Sciences and Veterinary Medicine of Cluj-Napoca, 3-5 Mănăştur St., 400372 Cluj-Napoca, Romania; rallucab@yahoo.com (S.R.I.), maria-ioana.socaciu@usamvcluj.ro (M.-I.S.); melinda.fogarasi@usamvcluj.ro (M.F.)

* Correspondence: cristina.semeniuc@usamvcluj.ro (C.A.S.); sonia.socaci@usamvcluj.ro (S.A.S.); Tel.: +40-264-596-384 (C.A.S. & S.A.S.)

Table S1. Centralizing table of extraction methods used.

Extraction method	Sample weight/Reagents and materials used	Reference
**Direct extraction method**	Sample weight: 1-4 g<br>Reagents: 0.9% sodium chloride solution, ethanol/hexane (1:5, *v*/*v*), sodium sulfate anhydrous, hexane	Weber et al., 1997
	Sample weight: 1 g<br>Reagents: 0.15 M sodium chloride solution (35 mL), ethanol (20 mL), *n*-hexane (100 mL), sodium sulfate anhydrous, 2-propanol (5 mL)	Mattila et al., 2000
	**For vegetable samples**<br>Sample weight: 2 g<br>Reagents: 0.15 M sodium chloride solution (5 mL), ethanol (18 mL), *n*-hexane (60 mL), 2-propanol (2 mL)<br>Sample filtration: 0.2 μm PTFE membrane filter, Lida	Mattila and Kumpulainen, 2001
	**For animal origin samples**<br>Sample weight: 7 g<br>Reagents: ethanol (18 mL), *n*-hexane (60 mL), 2-propanol (2 mL)<br>Sample filtration: 0.2 μm PTFE membrane filter, Lida	Mattila and Kumpulainen, 2001
	Sample weight: 10 mg<br>Reagents:<br>0.15 M sodium chloride solution (200 μL), ethanol (200 μL), *n*-hexane (1500 μL), 2-propanol (200 μL)	Purchas et al., 2004
	Sample weight: 1 g<br>Reagents: 0.15 M sodium chloride solution (5 mL), 0.1 M sodium dodecyl sulphate (5 mL), anhydrous ethanol (2 mL), hexane (10 mL), 2-propanol (1 mL)<br>Sample filtration: 0.45 μm PTFE membrane filter	Souchet and Laplante, 2007
	Sample weight: 2 g<br>Reagents: 0.15 M sodium chloride solution (5 mL), ethanol (18 mL), *n*-hexane (60 mL), 2-propanol (2 mL)<br>Sample filtration: 0.2 μm PTFE membrane filter	Ercan and El, 2011
	Sample weight: 1 g<br>Reagents: Hanks' balanced salt solution (20 mL), ethanol (6.5 mL), *n*-hexane (20 mL), 2-propanol (2.5 mL)<br>Sample filtration: 0.2 μm surfactant free cellulose acetate filters	Tobin et al., 2014
	Sample weight: 1 g<br>Reagents: 0.15 M sodium chloride solution (20 mL), ethanol (9 mL), *n*-hexane (30 mL), acetone (2.5 mL)	Román-Pizarro et al., 2017
	Sample weight: 0.05 g<br>Reagent: 2-propanol (7-12 mL)	Kubo et al., 2008
	**For oils extracted by enzymatic hydrolysis** and **oils extracted using SCO_2 600 g CO_2/h+5% EtOH**<br>Sample weight: unspecified amount<br>Reagent: 2-propanol (unspecified volume)	Laplante et al., 2009

Extraction method	Sample weight/Reagents and materials used	Reference
	Sample weight: 0.05-0.1 g<br>Reagents: 2-propanol (2-3 mL)	Stiff et al., 2011
	Sample weight: 10-60 mg<br>Reagents: 2-propanol (1500 μL), saline solution (150 μL), hexane (500 μL), methanol/ethanol/propanol (100:95:5, *v*/*v*/*v*) (60 μL)	Niklowitz et al., 2013
	Sample: 50 mg<br>Reagents: nitrogen-saturated ethanol/water (95:5, *v*/*v*) PBS solution (pH 6.5) (26.5 mL)	Li et al., 2016
**Ultrasonic extraction method**	Sample weight: 2.0 g<br>Reagents: liquid nitrogen, anhydrous ethanol (60 mL), hexane (unspecified volume)<br>Extraction time: 15 min	Zu et al., 2006
**Saponification-extraction method**	**For vegetable samples**<br>Sample weight: 0.2 g<br>Reagents: 2% ascorbic acid solution (2 mL), methanol (5 mL), aqueous potassium hydroxide solution (50 g KOH + 50 mL H_2O) (0.5 mL), 10% sodium chloride solution (7 mL), *n*-hexane (65 mL), 5% sodium chloride solution (20 mL), ethanol (5 mL), *n*-hexane/2-propanol (1:1, *v*/*v*) (2-5 mL)<br>Sample filtration: 0.2 μm PTFE membrane filter, Lida	Mattila and Kumpulainen, 2001
	**For animal samples**<br>Sample weight: 2 g<br>Reagents: 2% ascorbic acid solution (2 mL), methanol (5 mL), potassium hydroxide solution (50 g KOH + 50 mL H_2O) (0.5 mL), 10% sodium chloride solution (7 mL), *n*-hexane (65 mL), 5% sodium chloride solution (20 mL), ethanol (5 mL), *n*-hexane/2-propanol (3:7, *v*/*v*) (2 mL)<br>Sample filtration: 0.2 μm PTFE membrane filter, Lida	Mattila and Kumpulainen, 2001
	Sample weight: 1 g<br>Reagents: 5% aqueous pyrogallol solution (5 mL), 10% sodium hydroxide solution (5 mL), methanol (10 mL), 10% sodium chloride solution (10 mL), *n*-hexane (65 mL), 5% sodium chloride solution (20 mL), ethanol (5 mL), 2-propanol (1 mL)<br>Sample filtration: 0.45 μm PTFE syringe filter, Whatman	Pyo, 2010
	Sample weight: 50 g<br>Reagents for saponification: distilled water (50 g), pyrogallol (2.5 g), methanol (70 mL), 25% aqueous potassium hydroxide solution (25 mL), petroleum ether 40-60 °C (75 mL), sodium sulfate anhydrous, ethanol (5 mL)<br>Purification of the saponified extract over alumina (50 x 1 cm) column<br>Separation of CoQ10 from the purified extract on silica gel F_{254} glass plate (20 × 20 cm)	Al-Faraji and Shanshal, 2010
	Sample weight: 2 g<br>Reagents: 2% ascorbic acid solution (2 mL), methanol (5 mL), potassium hydroxide solution (50 g KOH + 50 mL H_2O) (0.5 mL), 10% sodium chlorine solution (7 mL), *n*-hexane (65 mL), 5% sodium chloride solution (20 mL), ethanol (5 mL), *n*-hexane/2-propanol (30:70, *v*/*v*) (2 mL)	Ercan and El, 2011

Extraction method	Sample weight/Reagents and materials used	Reference
	Sample weight: 0.5 g<br>Reagents: 60% potassium hydroxide solution (2 mL), 6% ethanolic pyrogallol solution (5 mL), 96% ethanol (2 mL), 1% sodium chloride solution (15 mL), hexane/ethyl acetate (9:1, *v*/*v*) (15 mL), 1% 2-propanol in *n*-hexane (2 mL)	Manzi and Durazzo, 2015
	Sample weight: 35 g<br>Reagents: 2 N ethanolic potassium hydroxide solution (35 mL), 1% ethanolic pyrogallol solution (20 mL), bidistilled water (105 mL), ethanol (10 mL), petroleum ether/diethyl ether (9:1, *v*/*v*) (70 mL), sodium sulfate anhydrous (5 g), 2-propanol (1 mL)<br>Sample filtration: 0.45 µm nylon syringe filter, Agilent Technologies	Mandrioli et al., 2018
**Solid-phase extraction (SPE)**	Sample weight: 0.25 g<br>Reagents: heptane (21.5 mL), heptane/ethyl ether (80:20, *v*/*v*) (34 mL), acetonitrile/tetrahydrofuran (90:10, *v*/*v*) (2 mL)<br>5 g of solid-phase extraction (SPE) cartridge with amino-propyl (NH_2) adsorbents, Varian	Rodríguez-Acuña et al., 2008
**Accelerated solvent extraction (ASE)**	Sample weight: 1 g<br>Reagents: Cleanert Alumina-N (3 g), absolute ethanol (19 mL)<br>Temperature: 80°C<br>Heat-up time: 5 min<br>Static time: 5 min<br>Flush volume: 60%<br>Purge time: 1 min<br>Number of cycles: 1<br>Cell volume: 10 mL<br>Total extraction time: 16-17 min/sample<br>Sample filtration: 0.45 µm nylon filter membrane, Phenomenex	Xue et al., 2012

16

Table S2. Centralizing table of quantification methods used.

Quantification method	Reference
HPLC with UV detection	Weber et al., 1997
	Purchas et al., 2004
	Al-Faraji and Shanshal, 2010
	Stiff et al., 2011
	Ercan and El, 2011
	Tobin et al., 2014
	Manzi and Durazzo, 2015
HPLC with DA detection	Mattila et al., 2000
	Mattila and Kumpulainen, 2001
	Souchet and Laplante, 2007
	Laplante et al., 2009
	Xue et al., 2012
	Román-Pizarro et al., 2017
	Mandrioli et al., 2018
HPLC with EC detection	Mattila et al., 2000
	Niklowitz et al., 2013
HPLC/ESI-MS detection	Pyo, 2010
HPLC/ESI-MS/MS detection	Zu et al., 2006
HPLC with AEC detection	Kubo et al., 2008
HPLC/APCI-MS detection	Rodríguez-Acuña et al., 2008
CEFS detection	Román-Pizarro et al., 2017
DPV using an electrochemical workstation	Li et al., 2016

HPLC-high-performance liquid chromatography; UV-ultraviolet; DA-Diode Array; EC-electrochemical; ESI-Electrospray ionization; MS-Mass spectrometry; AEC-amperometric electrochemical; APCI-atmospheric pressure chemical ionization; CEFS-Cary Eclipse fluorescence spectrometer; DPV-differential pulse voltammetry.

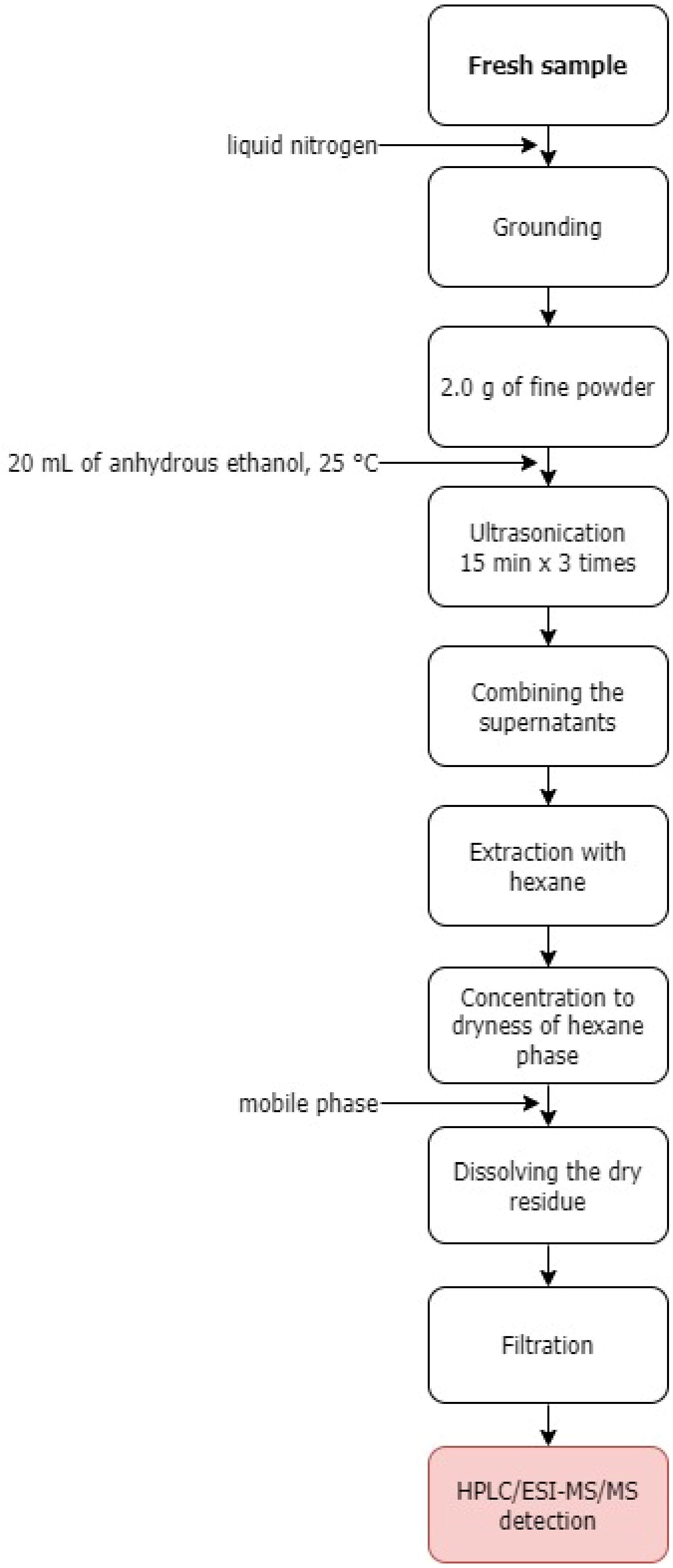

Figure S1. Workflow of the analytical method adapted from Zu et al., 2006.

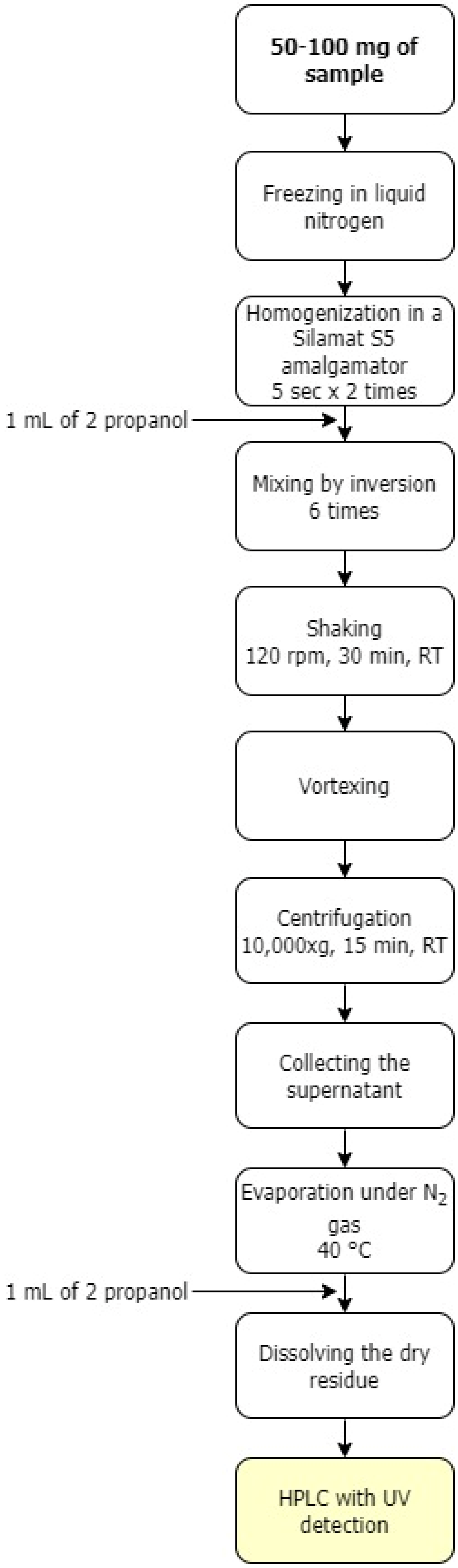

Figure S2. Workflow of the analytical method adapted from of Stiff et al., 2011.

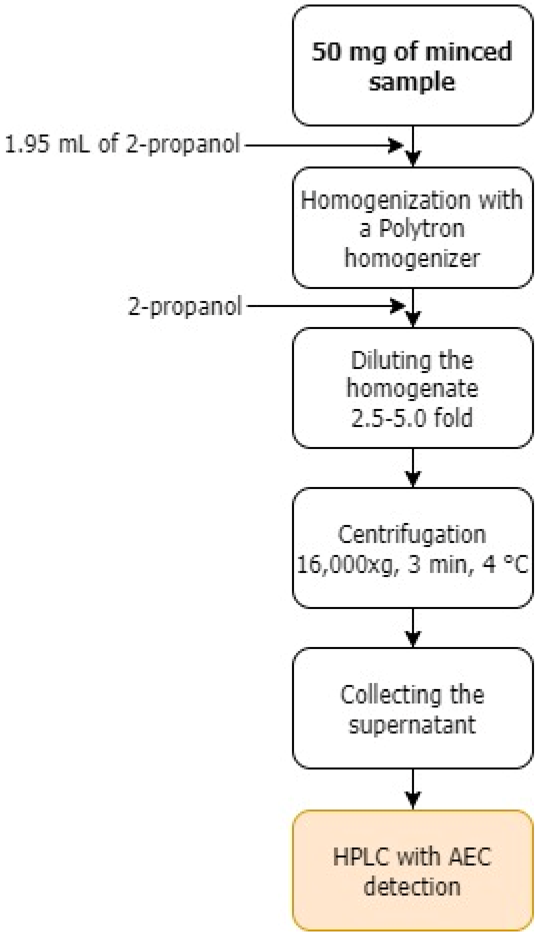

Figure S3. Workflow of the analytical method adapted from Kubo et al., 2008.

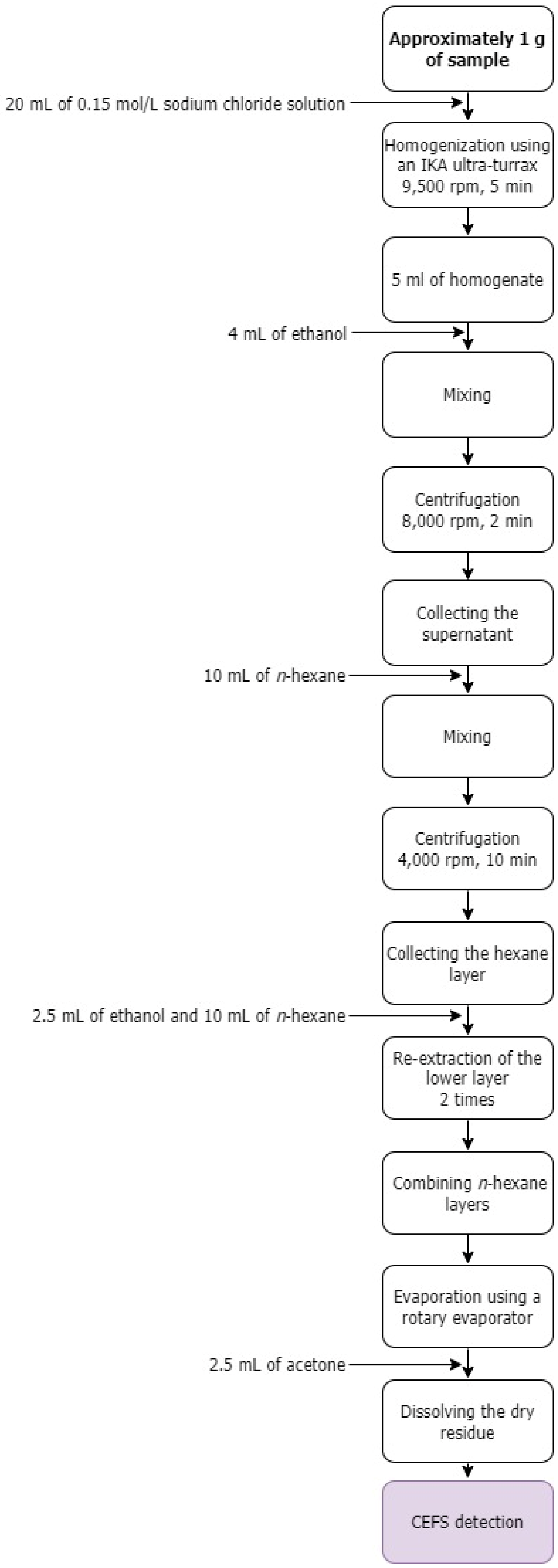

Figure S4. Workflow of the analytical method adapted from Román-Pizarro et al., 2017.

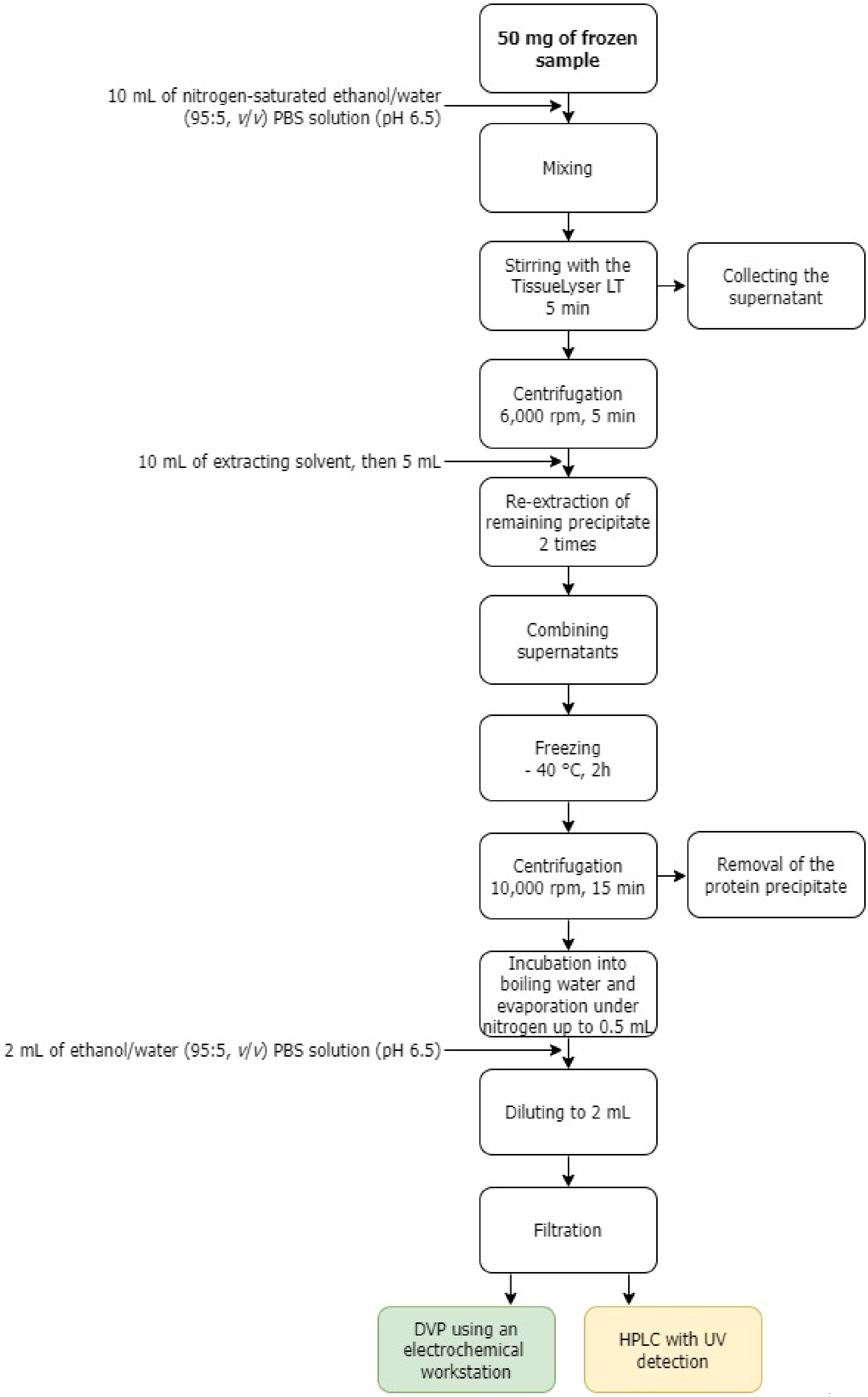

Figure S5. Workflow of the analytical method adapted from Li et al., 2016. 32

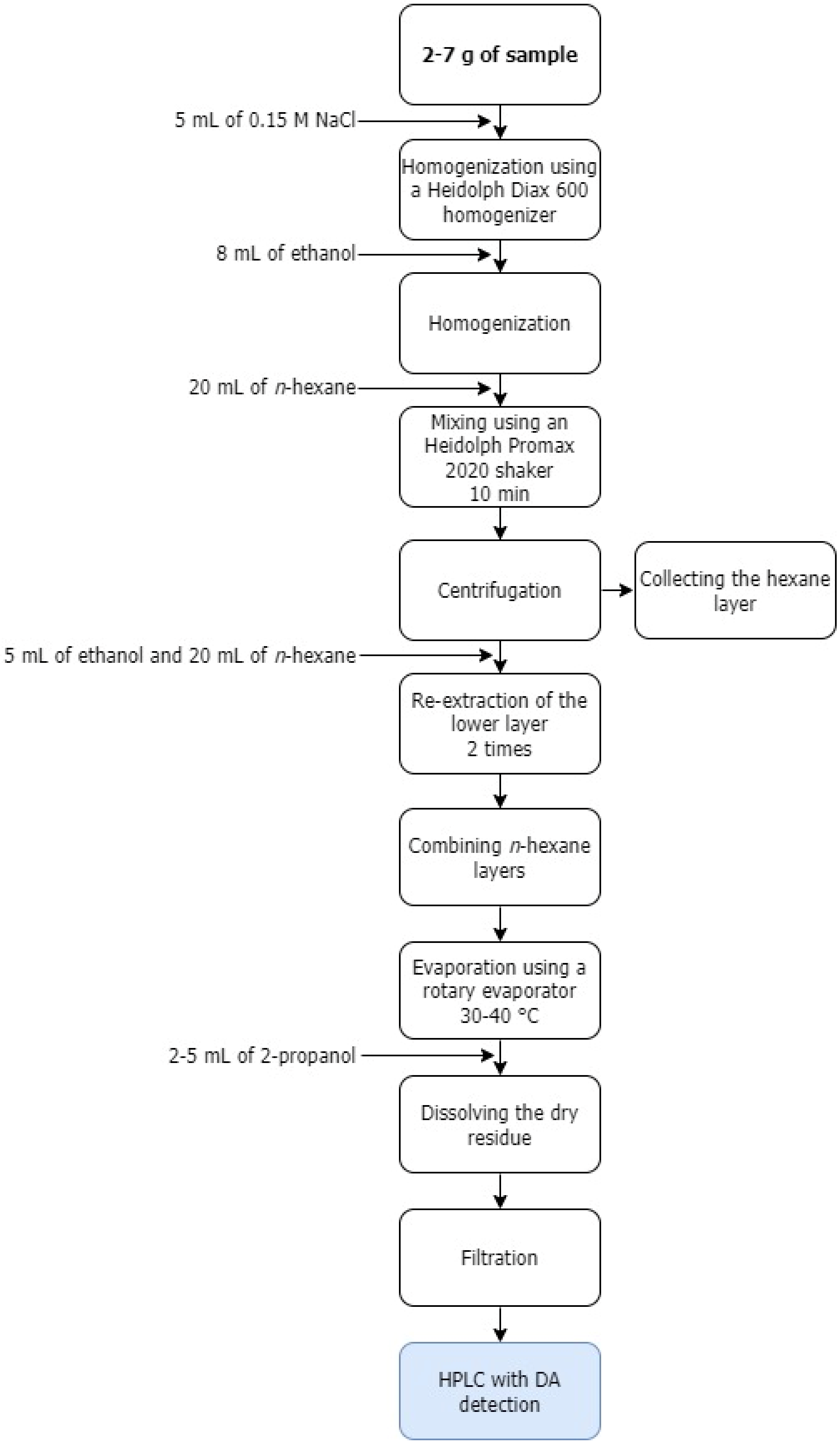

Figure S6. Workflow of the analytical method adapted from Mattila and Kumpulainen, 2001 (direct extraction method).

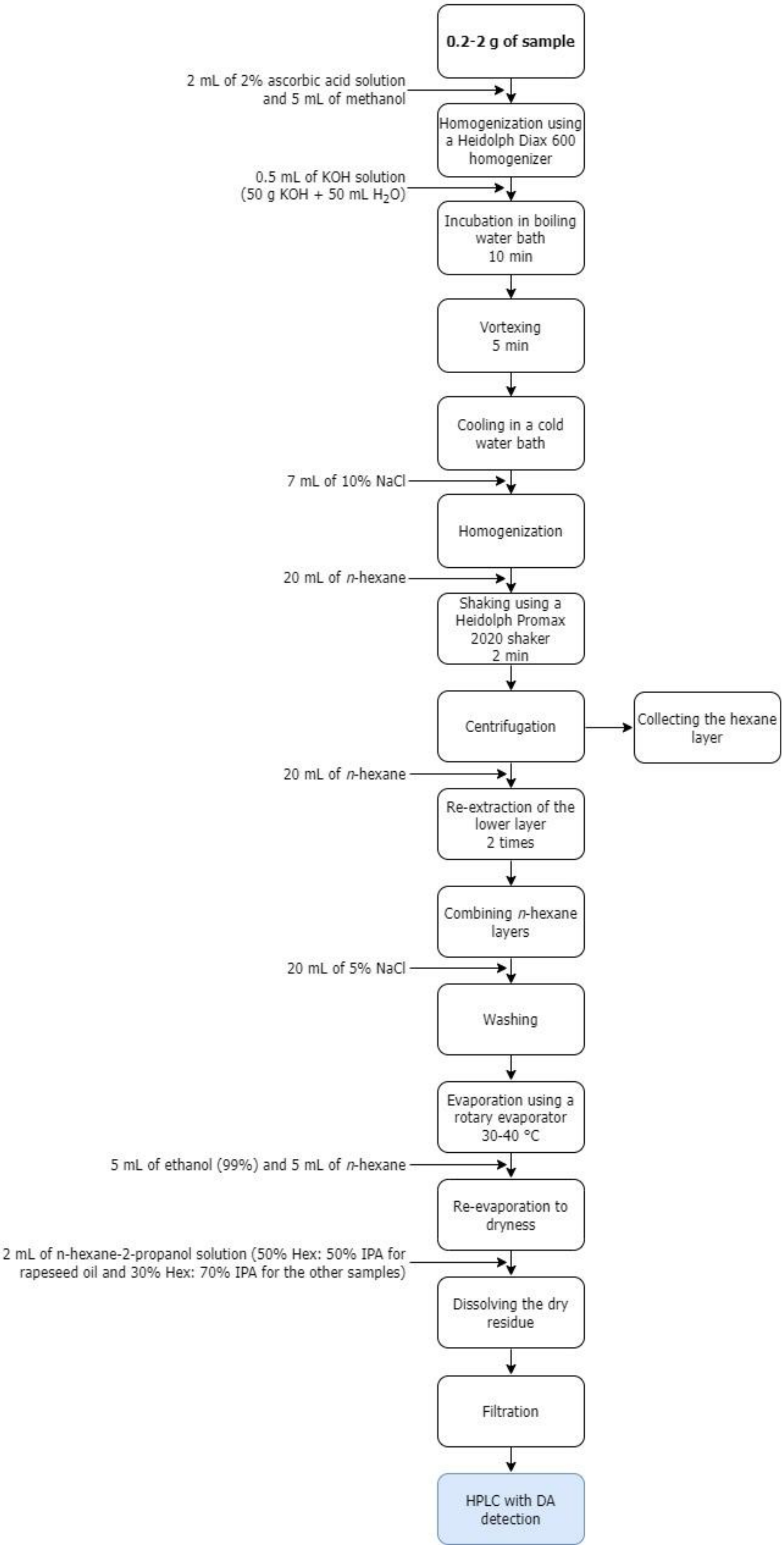

Figure S7. Workflow of the analytical method adapted from Mattila and Kumpulainen, 2001 (saponification-extraction method).

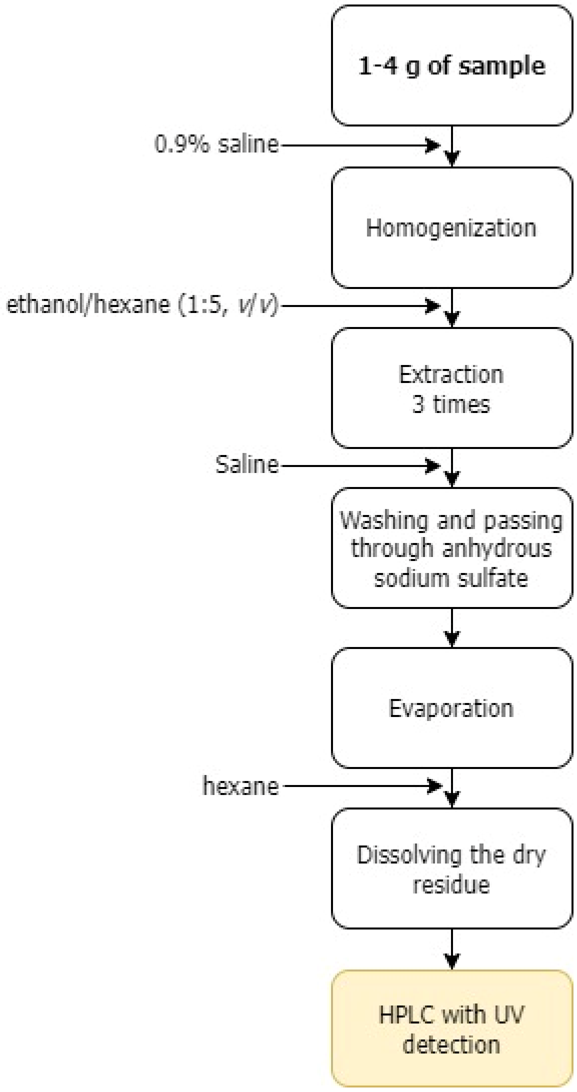

Figure S8. Workflow of the analytical method adapted from Weber et al., 1997.

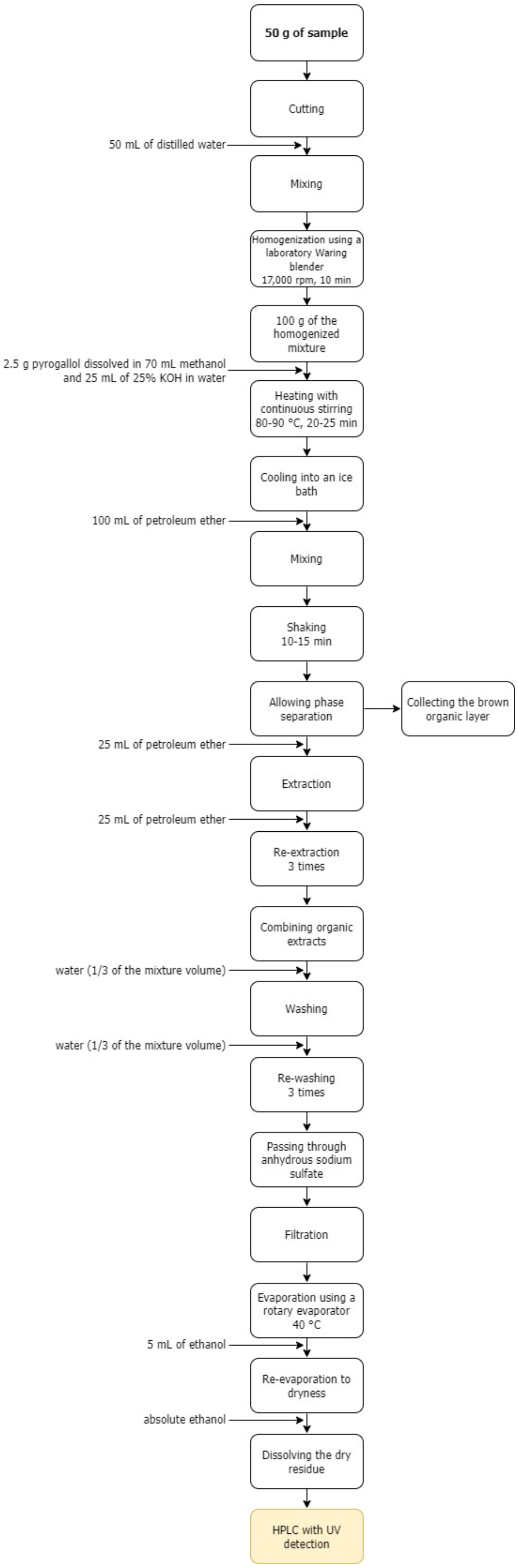

Figure S9. Workflow of the analytical method adapted from Al-Faraji and Shanshal, 2010.

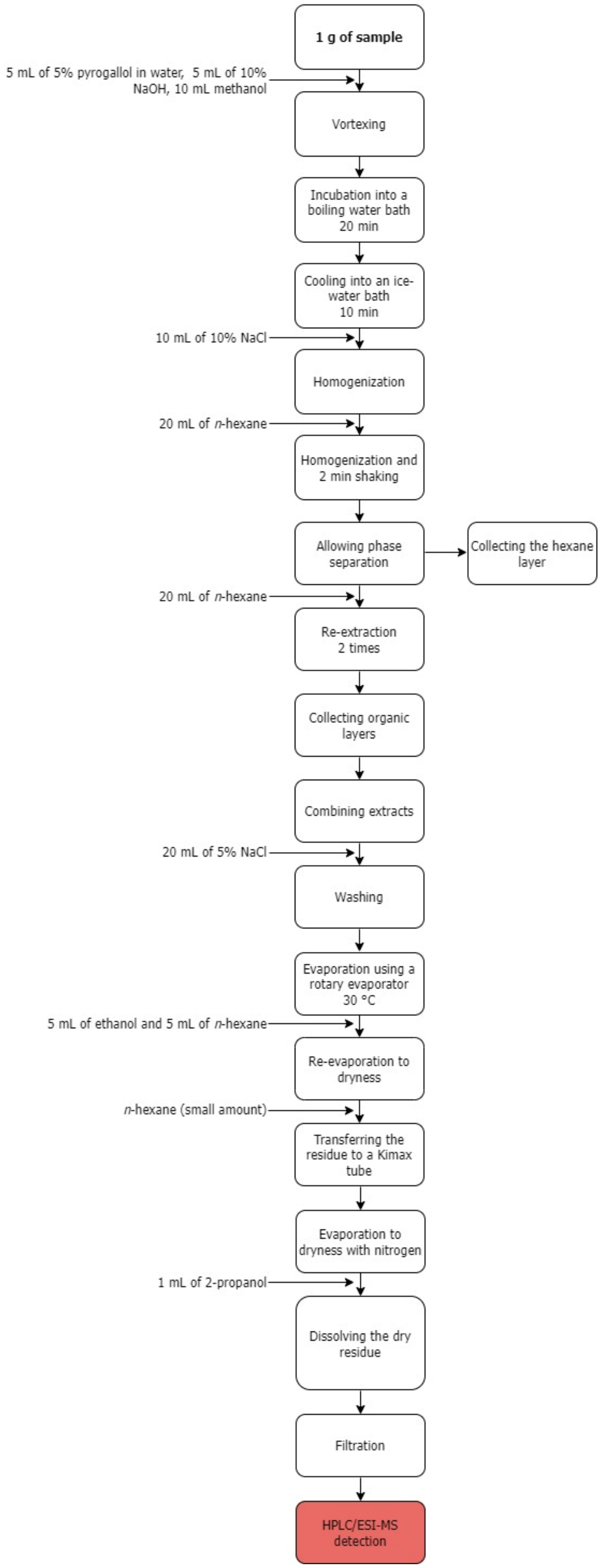

43

Figure S10. Workflow of the analytical method adapted from Pyo, 2010. 44

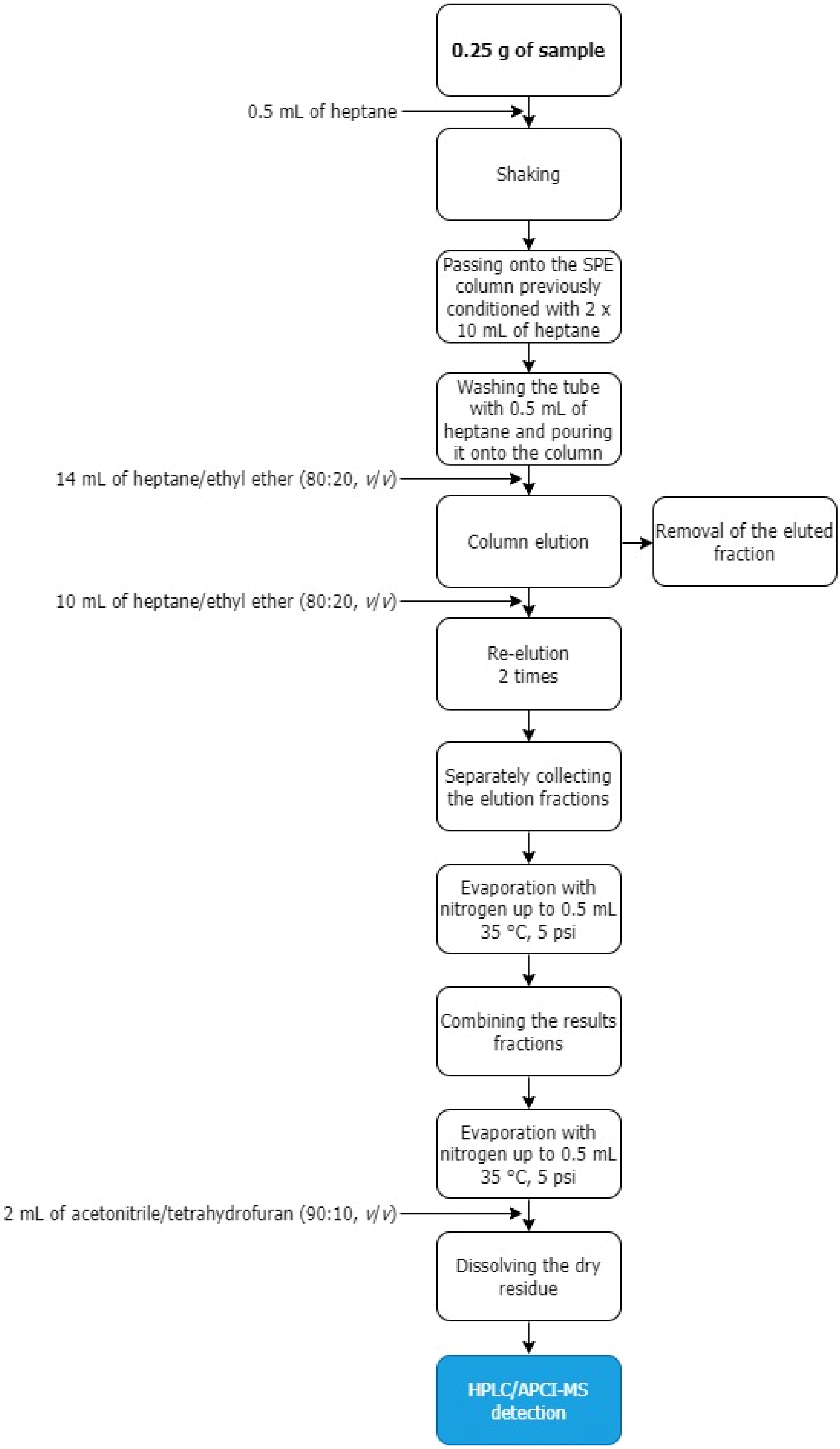

45

Figure S11. Workflow of the analytical method adapted from Rodríguez-Acuña et al., 2008. 46

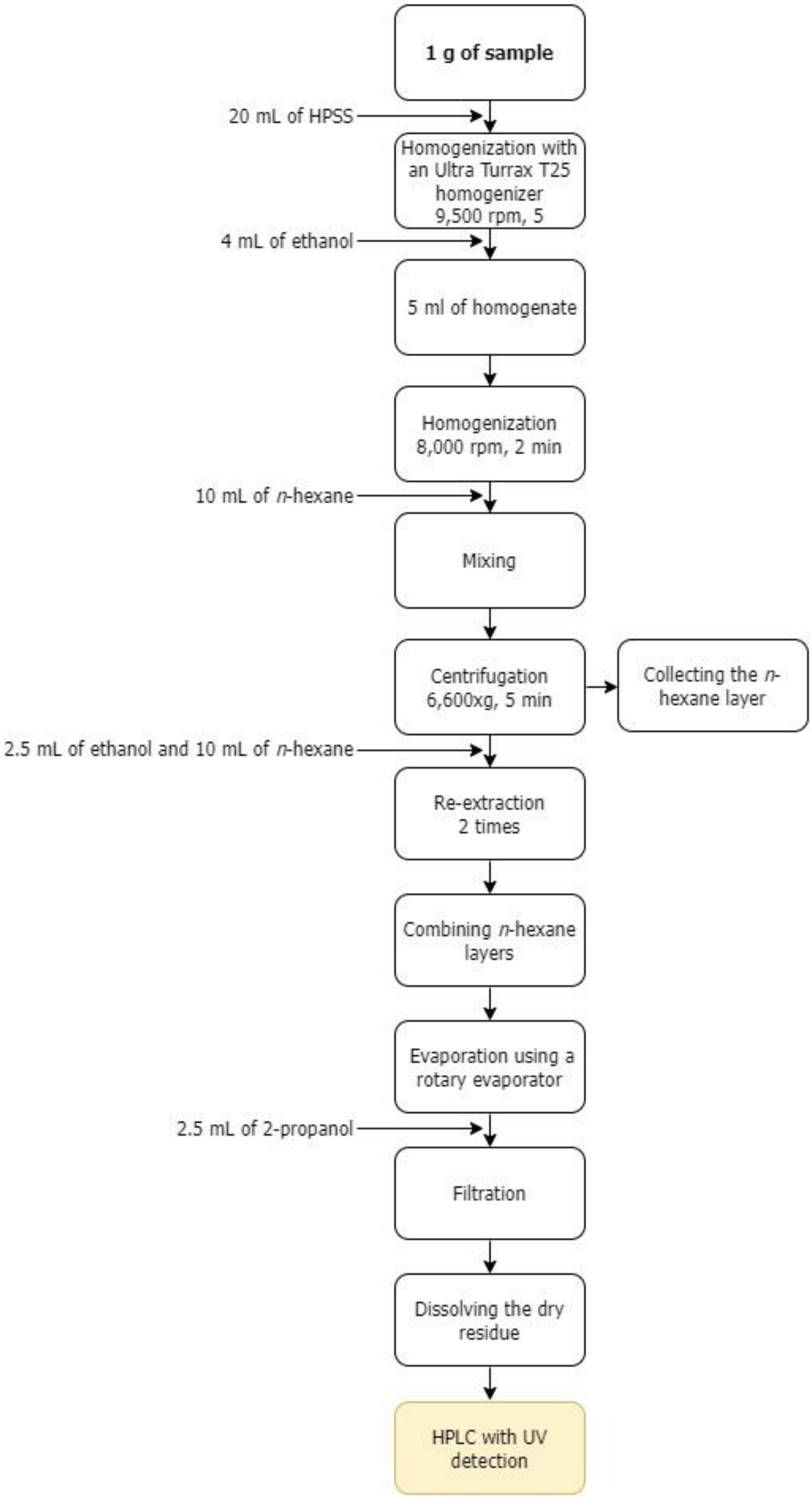

Figure S12. Workflow of the analytical method adapted from Tobin et al., 2014.

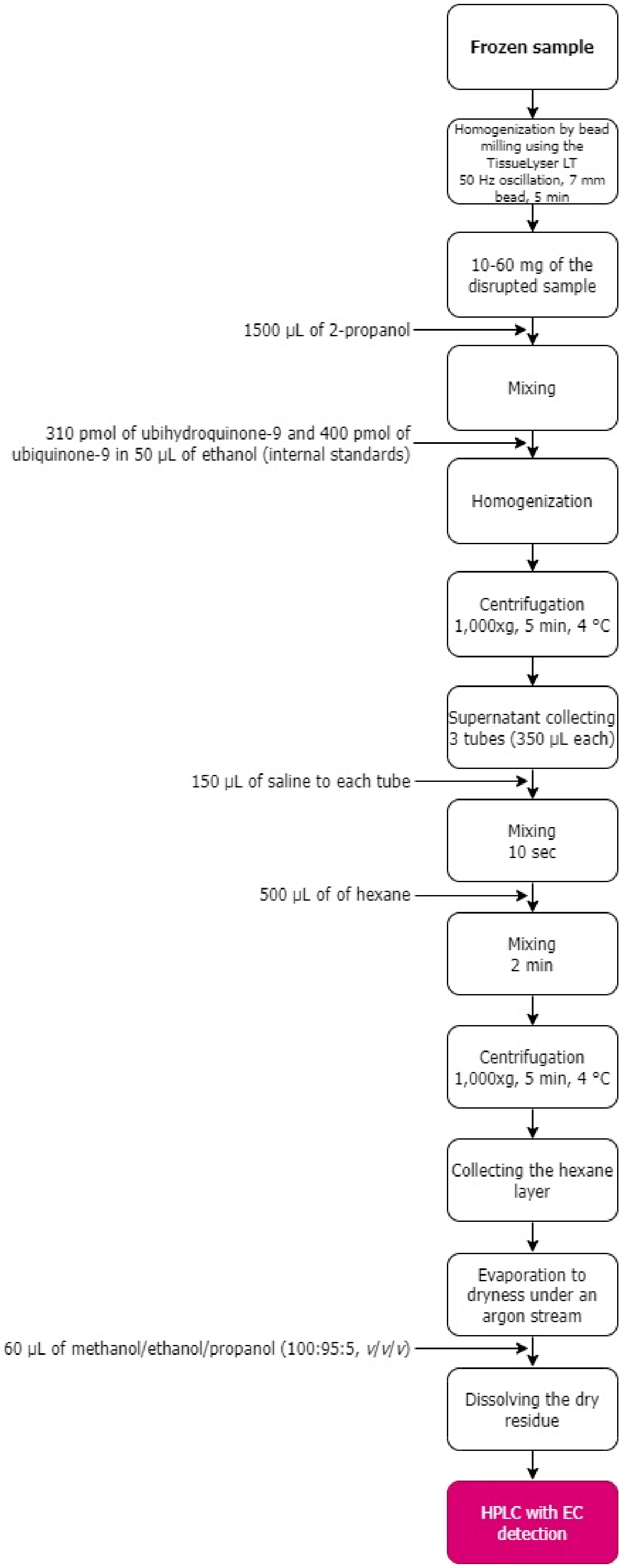

49
Figure S13. Workflow of the analytical method adapted from Niklowitz et al., 2013. 50

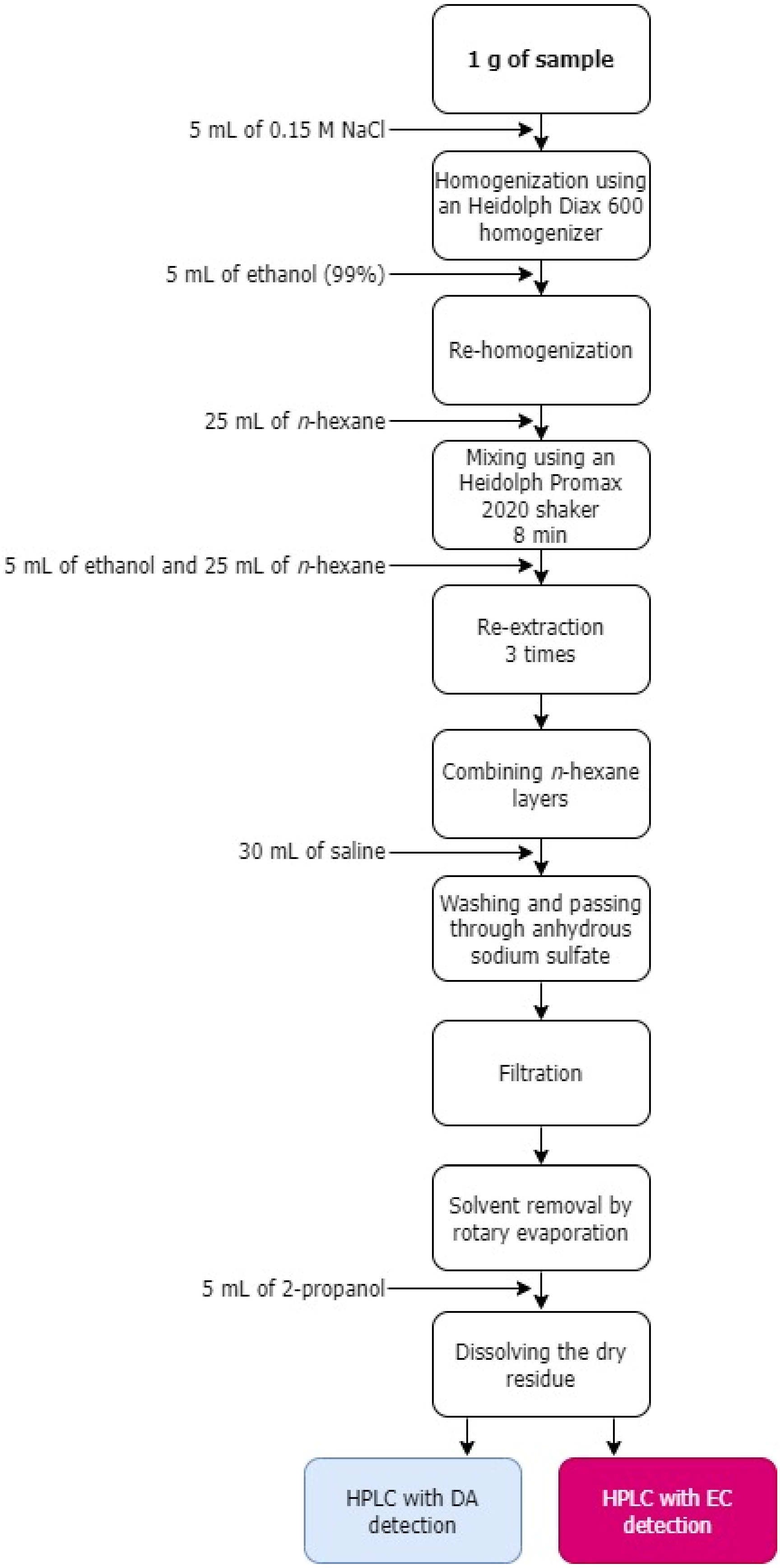

Figure S14. Workflow of the analytical method adapted from Mattila et al., 2000.

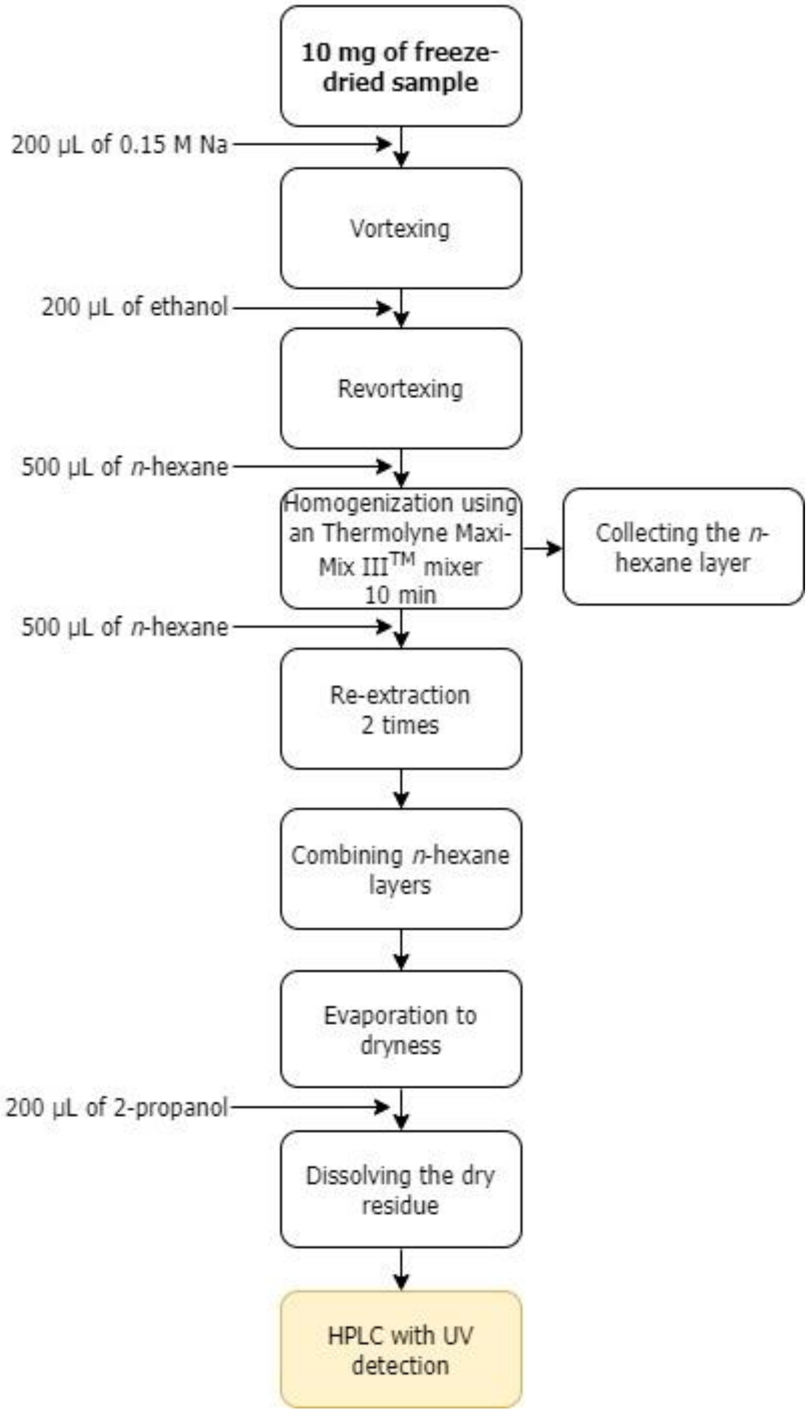

Figure S15. Workflow of the analytical method adapted from Purchas et al., 2004.

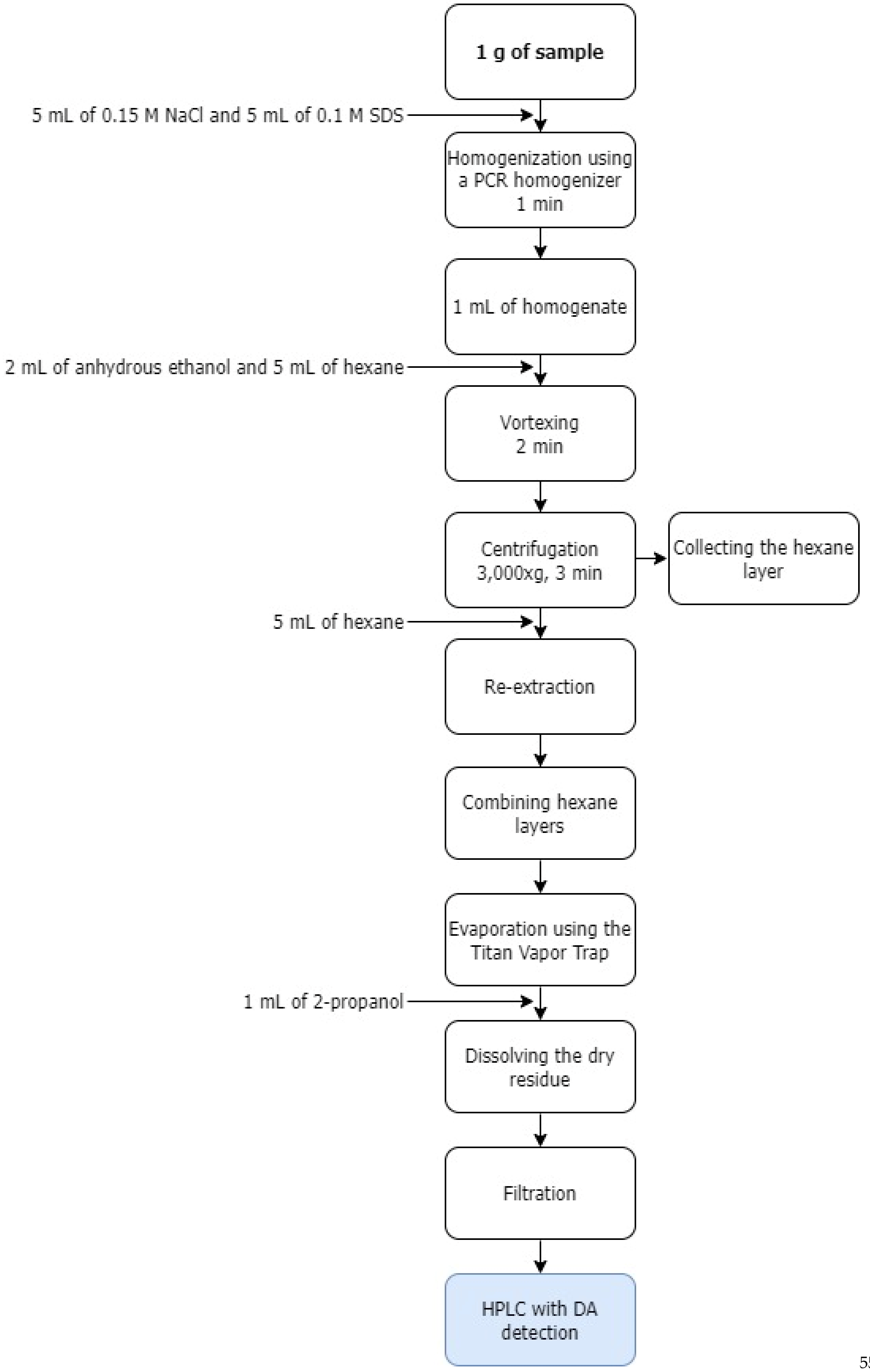

Figure S16. Workflow of the analytical method adapted from Souchet and Laplante, 2007.

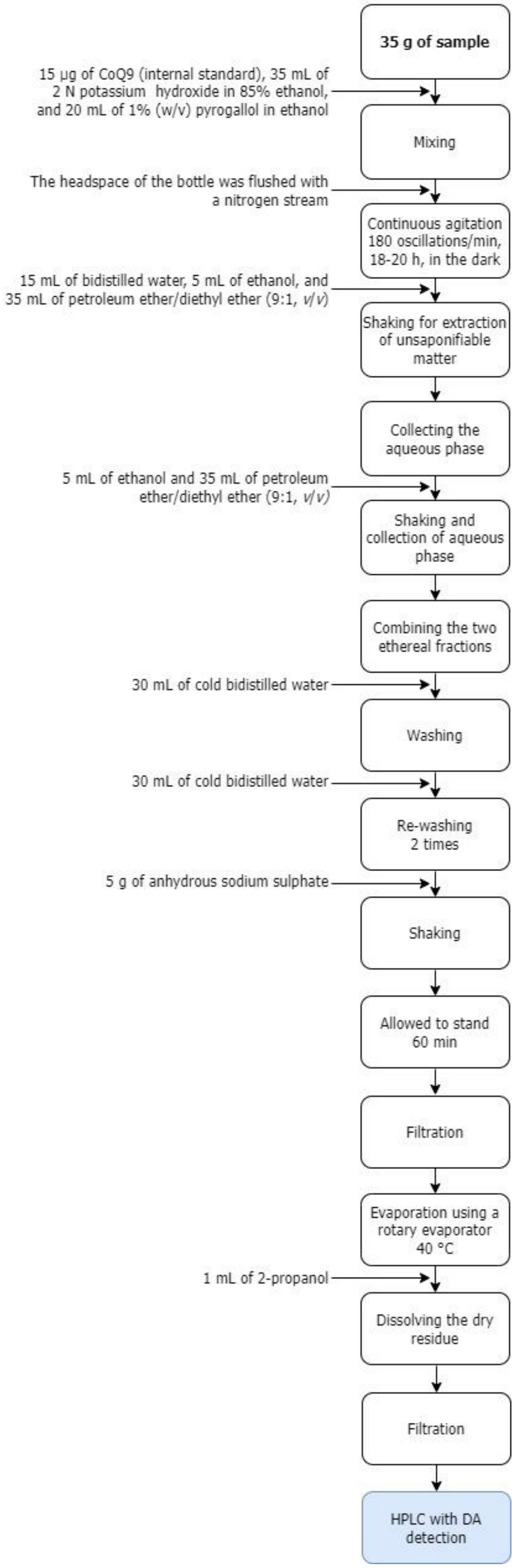

Figure S17. Workflow of the analytical method adapted from Mandrioli et al., 2018.

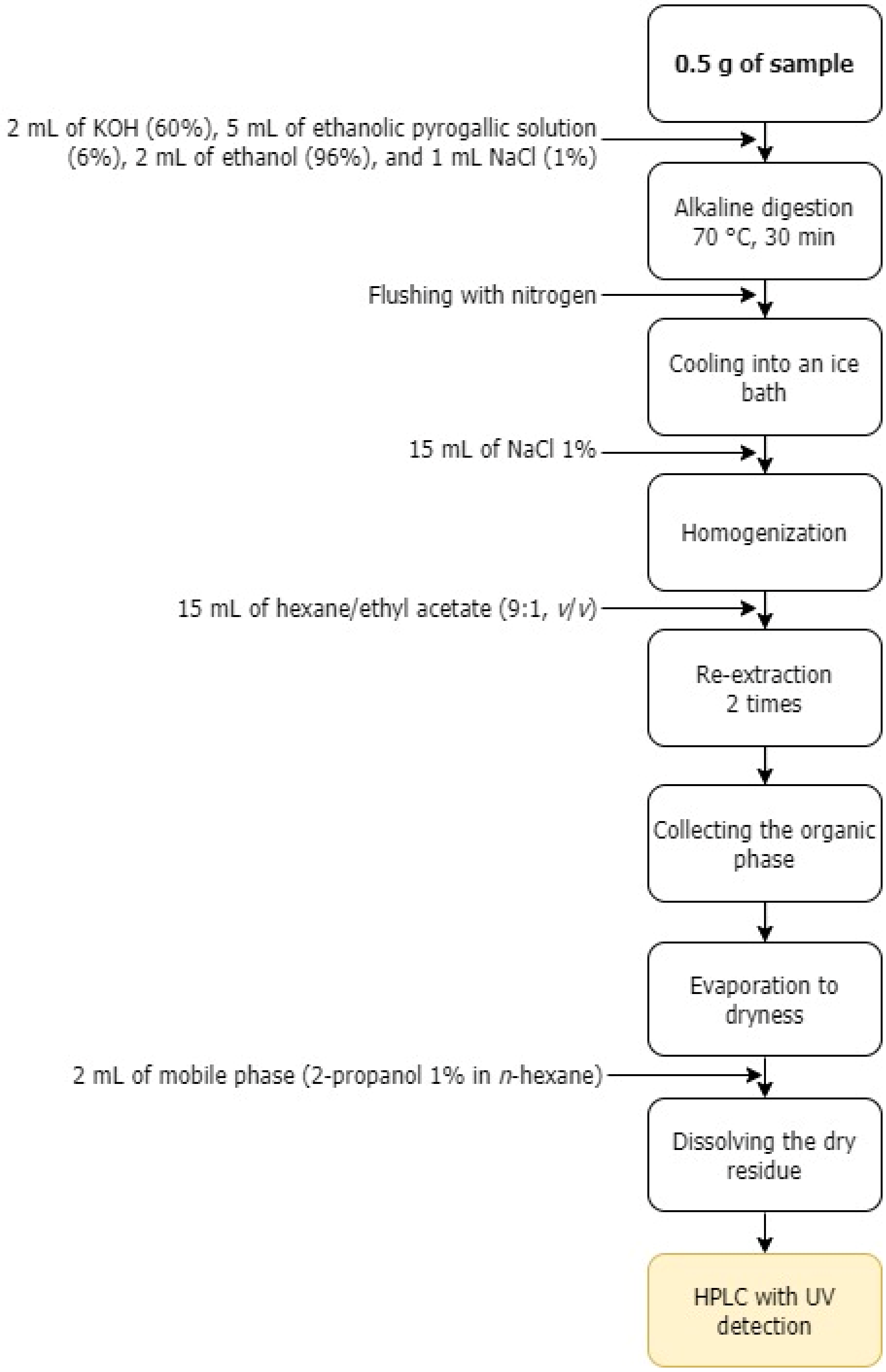

Figure S18. Workflow of the analytical method adapted from Manzi and Durazzo, 2015.

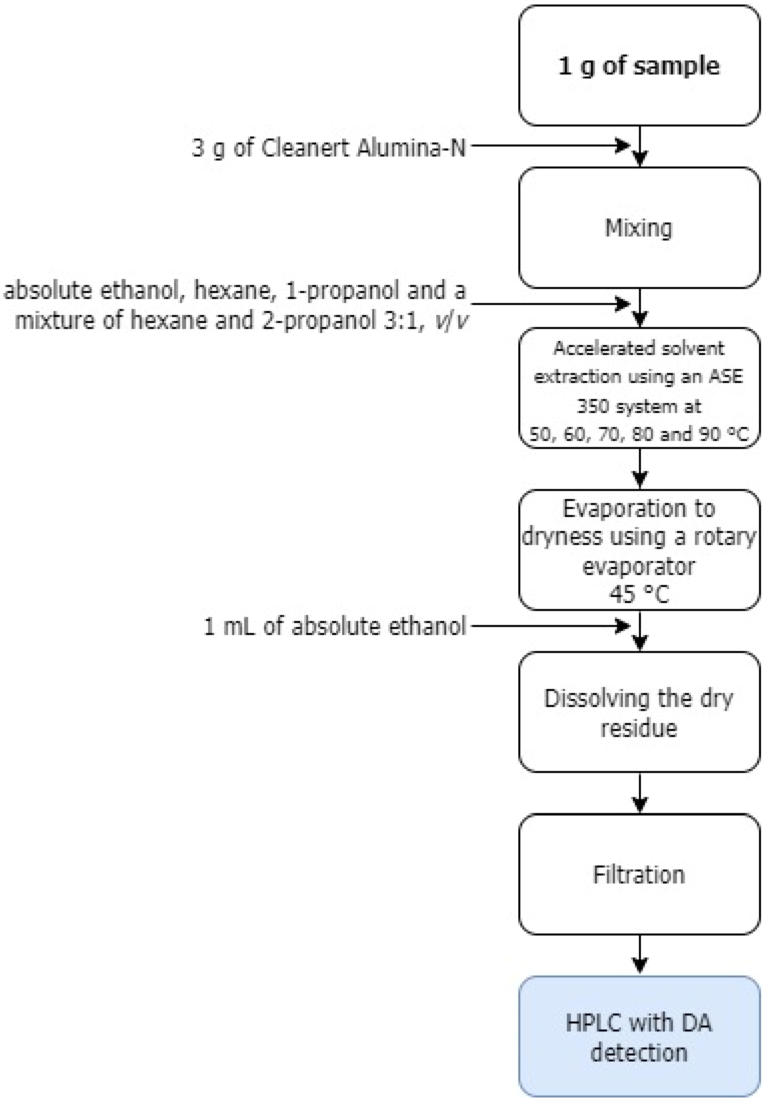

Figure S19. Workflow of the analytical method adapted from Xue et al., 2012.

References

1. Weber, C.; Bysted, A.; Hølmer, G. Coenzyme Q_{10} in the diet-daily intake and relative bioavailability. *Mol Aspects Med* **1997**, *18*, 251–254. https://doi.org/10.1016/S0098-2997(97)00003-4
2. Mattila, P.; Lehtonen, M.; Kumpulainen, J. Comparison of in-line connected diode array and electrochemical detectors in the high-performance liquid chromatographic analysis of coenzymes Q(9) and Q(10) in food materials. *JAFC* **2000**, *48*, 1229–1233. https://doi.org/10.1021/jf990581f
3. Mattila, P.; Kumpulainen, J. Coenzymes Q_9 and Q_{10}: contents in foods and dietary intake. *J Food Compo Anal* **2001**, *14*, 409–417. https://doi.org/10.1006/jfca.2000.0983
4. Purchas, R.W.; Rutherfurd, S.M.; Pearce, P.D.; Vather, R.; Wilkinson, B.H.P. Concentrations in beef and lamb of taurine, carnosine, coenzyme Q_{10}, and creatine. *Meat Sci* **2004**, *66*, 629–637. https://doi.org/10.1016/S0309-1740(03)00181-5
5. Souchet, N.; Laplante, S. Seasonal variation of Co-enzyme Q10 content in pelagic fish tissues from Eastern Quebec. *J Food Compo Anal* **2007**, *20*, 403–410. https://doi.org/10.1016/j.jfca.2006.09.004
6. Ercan, P.; El, S.N. Changes in content of coenzyme Q10 in beef muscle, beef liver and beef heart with cooking and *in vitro* digestion. *J Food Compos Anal* **2011**, *24*, 1136–1140. https://doi.org/10.1016/j.jfca.2011.05.002
7. Tobin, B.D.; O'Sullivan, M.G.; Hamill, R.; Kerry, J.P. Effect of cooking and *in vitro* digestion on the stability of co-enzyme Q10 in processed meat products. *Food Chem* **2014,** *150*, 187–192. https://doi.org/10.1016/j.foodchem.2013.10.138
8. Román-Pizarro, V.; Fernández-Romero, J.M.; Gómez-Hens, A. Automatic determination of coenzyme Q10 in food using cresyl violet encapsulated into magnetoliposomes. *Food Chem* **2017**, *221*, 864–870. https://doi.org/10.1016/j.foodchem.2016.11.085
9. Kubo, H.; Fujii, K.; Kawabe, T.; Matsumoto, S.; Kishida, H.; Hosoe, K. Food content of ubiquinol-10 and ubiquinone-10 in the Japanese diet. *J Food Compo Anal* **2008**, *21*, 199–210. https://doi.org/10.1016/j.jfca.2007.10.003
10. Laplante, S.; Souchet, N.; Bryl, P. Comparison of low-temperature processes for oil and coenzyme Q10 extraction from mackerel and herring. *Eur J Lipid Sci Technol* **2009**, *111*, 135–141. https://doi.org/10.1002/ejlt.200800133
11. Stiff, M.R.; Weissinger, A.K.; Danehower, D.A. Analysis of CoQ_{10} in cultivated tobacco by a high-performance liquid chromatography–ultraviolet method. *J Agric Food Chem* **2011**, *59*, 9054–9058. https://doi.org/10.1021/jf201130z
12. Niklowitz, P.; Döring, F.; Paulussen, M.; Menke, T. Determination of coenzyme Q10 tissue status via high-performance liquid chromatography with electrochemical detection in swine tissues (*Sus scrofa domestica*). *Anal Biochem* **2013,** *437*, 88–94. https://doi.org/10.1016/j.ab.2013.02.015
13. Li, D.; Deng, W.; Xu, H.; Sun, Y.; Wang, Y.; Chen, S.; Ding, X. Electrochemical investigation of coenzyme Q10 on silver electrode in ethanol aqueous solution and its determination using differential pulse voltammetry. *J Lab Autom* **2016**, *21*, 579–589. https://doi.org/10.1177/2211068216644442
14. Zu, Y.; Zhao, C.; Li, C.; Zhang, L. A rapid and sensitive LC-MS/MS method for determination of coenzyme Q_{10} in tobacco (*Nicotiana tabacum* L.) leaves. *J Sep Sci* **2006**, *29*, 1607–1612. https://doi.org/10.1002/jssc.200600047
15. Pyo, Y.H. Coenzyme Q_{10} and Q_9 contents in 6 commercial vegetable oils and their average daily intakes in Korea. *Food Sci Biotechnol* **2010**, *19*, 837–841. https://doi.org/10.1007/s10068-010-0118-7
16. Al-Faraji, G.; Shanshal, M. Determination of Ubiquinone,10 in ten different sorts of Iraqi dates "*Phoenix dactylifera*" at different stages of fruit maturation. *JJC* **2010**, *5*, 389–400.
17. Manzi, P.; Durazzo, A. Rapid determination of coenzyme Q10 in cheese using high-performance liquid chromatography. *Dairy Sci Technol* **2015**, *95*, 533–539. https://doi.org/10.1007/s13594-015-0222-9
18. Mandrioli, M.; Semeniuc, C.A.; Boselli, E.; Rodriguez-Estrada, M.T. Ubiquinone in Italian high-quality raw cow milk. *Ital J Food Sci* **2018**, *30*, 144–155. https://doi.org/10.14674/IJFS-871
19. Rodríguez-Acuña, R.; Brenne, E.; Lacoste, F. Determination of coenzyme Q10 and Q9 in vegetable oils. *J Agric Food Chem* **2008**, *56*, 6241–6245. https://doi.org/10.1021/jf800103e
20. Xue, X.; Zhao, J.; Chen, L.; Zhou, J.; Yue, B.; Li, Y.; Wu, L.; Liu, F. Analysis of coenzyme Q10 in bee pollen using online cleanup by accelerated solvent extraction and high performance liquid chromatography. *Food Chem* **2012**, *133*, 573–578. https://doi.org/10.1016/j.foodchem.2011.12.085
